# Decision Theory versus Conventional Statistics for Personalized Therapy of Breast Cancer

**DOI:** 10.3390/jpm12040570

**Published:** 2022-04-02

**Authors:** Michael Kenn, Rudolf Karch, Dan Cacsire Castillo-Tong, Christian F. Singer, Heinz Koelbl, Wolfgang Schreiner

**Affiliations:** 1 Section of Biosimulation and Bioinformatics, Center for Medical Statistics, Informatics and Intelligent Systems (CeMSIIS), Medical University of Vienna, Spitalgasse 23, 1090 Vienna, Austria; michael.kenn@meduniwien.ac.at (M.K.); rudolf.karch@meduniwien.ac.at (R.K.); 2 Translational Gynecology Group, Department of Obstetrics and Gynecology Comprehensive Cancer Center, Medical University of Vienna, Waehringer Guertel 18-20, 1090 Vienna, Austria; dan.cacsire-castillo@meduniwien.ac.at (D.C.C.-T.); christian.singer@meduniwien.ac.at (C.F.S.); 3 Department of General Gynecology and Gynecologic Oncology, Medical University of Vienna, Waehringer Guertel 18-20, 1090 Vienna, Austria; heinz.koelbl@meduniwien.ac.at

**Keywords:** biomarkers, decision theory, gene expression, breast cancer, receptor status, precision medicine, personalized medicine, data science, mathematical oncology

## Abstract

Estrogen and progesterone receptors being present or not represents one of the most important biomarkers for therapy selection in breast cancer patients. Conventional measurement by immunohistochemistry (IHC) involves errors, and numerous attempts have been made to increase precision by additional information from gene expression. This raises the question of how to fuse information, in particular, if there is disagreement. It is the primary domain of Dempster–Shafer decision theory (DST) to deal with contradicting evidence on the same item (here: receptor status), obtained through different techniques. DST is widely used in technical settings, such as self-driving cars and aviation, and is also promising to deliver significant advantages in medicine. Using data from breast cancer patients already presented in previous work, we focus on comparing DST with classical statistics in this work, to pave the way for its application in medicine. First, we explain how DST not only considers probabilities (a single number per sample), but also incorporates uncertainty in a concept of ‘evidence’ (two numbers per sample). This allows for very powerful displays of patient data in so-called ternary plots, a novel and crucial advantage for medical interpretation. Results are obtained according to conventional statistics (ODDS) and, in parallel, according to DST. Agreement and differences are evaluated, and the particular merits of DST discussed. The presented application demonstrates how decision theory introduces new levels of confidence in diagnoses derived from medical data.

## 1. Introduction

### 1.1. Biomarkers: A Cornerstone of Personalized Medicine for Breast Cancer

Biomarkers gain importance in selecting treatments optimized and personalized to the individual needs of patients, as envisaged in personalized medicine [1,2,3,4]. Breast cancer treatment has also benefited from personalized medicine, via molecular subtyping [5,6,7,8], the Gene expression Grade Index [9], pathway analysis and networks [10,11,12,13,14] and a plethora of expression signatures [15,16,17,18,19,20,21,22], dedicated to special questions and issues. Well-known indicators supporting therapy selection for breast cancer are PAM50 [23,24], PREDICT [25], and the Genomic Grade Index [26].

For breast cancer, the HER2-status (human epidermal growth factor receptor 2) of a patient is one of the most important prognostic factors [27,28]. The majority of patients (75–85%) are HER2-negative and, therefore, have a much better prognosis. In this work, we focus on these and disregard HER2-positive ones, in order to increase the homogeneity of data and precision of predictions. HER2 is routinely determined by immunohistochemistry (IHC). Numerous studies covered the significance and accuracy of estimates [29,30,31,32,33,34,35,36]. In a previous paper [37], we described, in detail, how to select patients who are HER2 negative to a high degree of confidence by using the ODDS method. We draw on the very same database in the current work.

Among HER2-negative patients, the hormone receptor status of estrogen (ER) and progesterone (PGR) are of focal importance. Clinically, patients are considered receptor-positive if at least one of both receptors (ER or PGR) is found positive. Since hormone receptors play a role in promoting cancer, hormone therapy has to be part of an effective treatment. In patients without metastases, hormone therapy may even render chemotherapy unnecessary.

However, if the receptor status is accidentally estimated as false positive, hormone treatment will not work and the patient may be deprived of life-saving chemotherapy. Therefore, numerous studies have evaluated the quality of receptor assessment [38,39] and revealed a possible rate of misclassification of 10% to 20% [40,41,42]. Although standard operation procedures have been implemented [40,43,44], the improvement of precision is still desirable [45].

One possibility is adding information from gene expression. Some approaches merely used visual inspection to set cut-points between positive and negative [46], some used frequency distributions of expression values [42,47], random sampling [48] or fuzzy rules [49] and other methodological advances in gene expression analysis [50,51,52,53,54,55,56,57].

We have elaborated and improved the above approaches [58,59] by introducing **Dempster–Shafer** decision Theory (DST) [60] into personalized medicine [37]. This is promising, since decision theory has demonstrated its benefit in many technical settings, such as self-driving cars [61,62,63], observing a driver’s vigilance [62], aviation security [64,65] and also in some medical applications, such as image-based decisions [66], diagnosis of prostate [67] and breast cancer [68]. The specific merit of DST is the capability to handle unclear or contradicting information obtained from different sources about the same issue in question (e.g., receptor status). DST is able to combine multifactor and even diverging evidence, according to exact algorithms, with the potential to increase the precision of medical decisions.

In the present work, we draw on data from our previous paper [37] and elaborate on the key differences between classical statistics and DST. Ternary plots are introduced for the interpretation of probabilities, in case of contradicting evidence—a potent concept from technics is tailored to the needs of personalized medicine.

### 1.2. Basic Concepts of Decision Theory for Hormone Receptor Status Assessment

DST is a general theory for reasoning with uncertainty [69]. It starts with the outcome of measuring processes, rather than from ‘true values’ present in reality, as conventional statistics does. We present DST in a mode with only two statuses, ‘+’ and ‘−’. This simplifies the formalism significantly.

Suppose some continuous variable, *d*, is being measured (e.g., *d* ≙ deepness of IHC-staining). Conventional statistics would derive a single number from *d*, the probability *p* for the receptor being positive, given the measured value of *d*. Consequently, 1 − *p* would be the probability for being negative.

DST provides *two* numbers to characterize possible predictions based on measuring *d*:

- The belief *α*(*d*), gives the probability (weight) that, upon measuring this particular value of *d*, the prediction ‘positive’ can be made based on the quality of measurement (classification ‘with full right’).
- The uncertainty *θ*(*d*), characterizing the probability (weight), that the prediction ‘positive’ could root in chance and not in quality of measurement. Belief and uncertainty taken together yield the total probability (termed ‘plausibility’) to obtain the prediction ‘positive’, given the measured value of *d* (*α* + *θ* = *pl*).
- Finally, a third number can be computed from belief and uncertainty, the probability *β*(*d*) for yielding the prediction ‘negative’ by quality of measurement, given *d*. We always have: *α* + *θ* + *β* = 1; hence, *β* can be computed from *α* and *θ*.

In the special case of only two statuses, as considered here, the triple (*α*, *β*, *θ*) is equivalent to a piece of ‘evidence’. DST not only yields probabilities for a positive versus negative outcome, but, additionally, incorporates the uncertainty of prediction [70]. This represents a significant surplus and motivates its introduction into personalized medicine.

**Figure 1:**
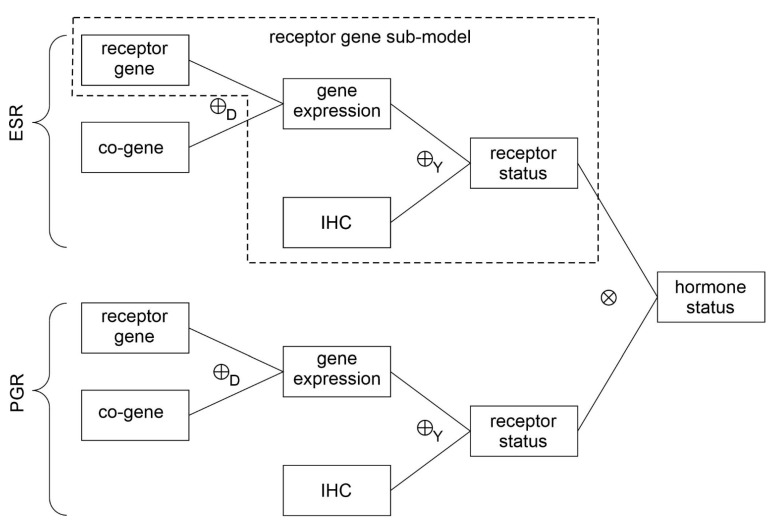
Combining evidence. For estrogen (ESR) and progesterone (PGR) similar procedures are applied to obtain receptor statuses. First evidence for receptor gene and co-gene are combined by Dempster rule (⊕_D_) and the result is combined with evidence for IHC by Yager rule (⊕_Y_). Finally, receptor statuses for estrogen and progesterone are combined (⊗) to obtain hormone receptor status. For detailed illustration of evidence combination (Section 2.2.1, Section 2.2.2, Section 2.2.3 and Section 2.2.4 and Appendix A.6) we start with a ‘receptor gene sub-model’ indicated by the dashed polygon.

A second advantage of DST is its capability of merging evidence from different sources (see also Figure 1 and the graphical abstract). In our case these will be:

- For estrogen (ER)
  ○ Receptor status predicted from expression of the receptor gene
  ○ Receptor status predicted from expression of a co-gene
    ▪ Combining above evidence by Dempster evidence combination rule ⊕_D_
  ○ Receptor status predicted from IHC
    ▪ Combining evidence from gene expression and IHC by Yager evidence combination rule ⊕_Y_

- For progesterone (PGR)
  ○ Receptor status predicted from expression of the receptor gene
  ○ Receptor status predicted from expression of a co-gene
    ▪ Combining above evidence by Dempster evidence combination rule ⊕_D_
  ○ Receptor status predicted from IHC
    ▪ Combining evidence from gene expression and IHC by Yager evidence combination rule ⊕_Y_

- Hormone receptor status is finally obtained by combining the statuses of estrogen and progesterone using a multiplicative combination rule ⊗

Details and references regarding the above workflow will be given in the following and are illustrated in Figure 1.

### 1.3. Ternary Plots: A Novel View on Evidence in Personalized Medicine

Another important point of the current work is to introduce so-called ‘ternary plots’, as a tailored tool to display not only probabilities, but also the uncertainties involved. We will evolve the framework step by step, to contrast conventional statistics against DST, thereby featuring the surpluses of DST.

## 2. Materials and Methods

### 2.1. Preliminaries on the Structure of the Methods’ Section

For the sake of readability and to present this paper self-contained, data cleansing and the concept of responsibility functions are not expanded in the methods sections but recapitulated in the Appendix A.1, Appendix A.2 and Appendix A.3. These computational procedures equal those detailed in our previous work [37]. Moreover, the methods’ Section 2.2.1, Section 2.2.2, Section 2.2.3 and Section 2.2.4 as well as Appendix A.6 are restricted to a single gene for didactical reasons (see the ‘receptor gene sub-model’ in Figure 1). In a thorough primary introduction, it seems important to demonstrate in detail how evidence from gene expression and IHC intermingle and eventually produce remarkable patterns in the data. These patterns demonstrate the dominant impact of IHC status on final predictions. Figures in Section 2.2.1, Section 2.2.2, Section 2.2.3 and Section 2.2.4 and Appendix A.6 exemplify intricate features on data for estrogen but methods are general and identically apply to all other parts of the ‘full’ model. 

In the final sections of methods (Section 2.3), we return to the full model shown in Figure 1, including co-genes. Line-like patterns are smeared out and do not remain visible as clearly as in the single-gene case. This full model was used to obtain the actual results for the patient cohort (Section 3).

### 2.2. Estrogen Receptor Gene Sub-Model

#### 2.2.1. Logistic Regression as Prerequisite

**Figure 2:**
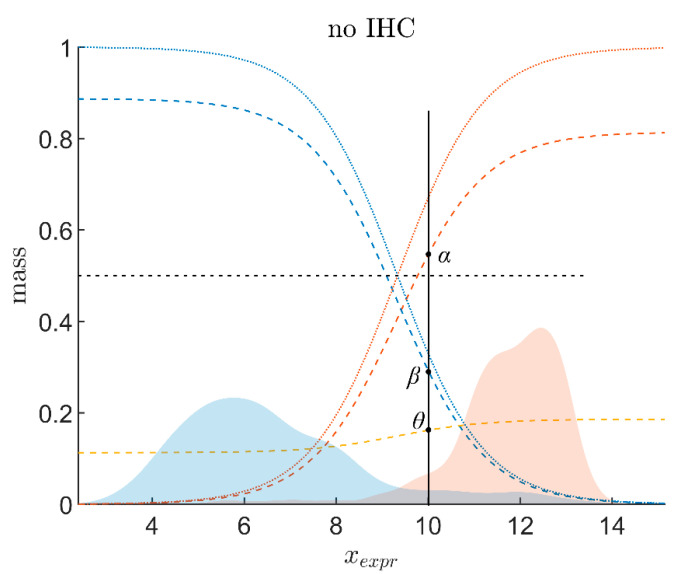
Logistic regression to obtain responsibility functions for decision theory evidence (data for estrogen, gene ESR1). Red-shaded area: distribution of gene expression for receptor positive (according to IHC) computed from density kernel estimates [71,72,73]. Blue shaded area: gene expression for negative IHC. IHC receptor status (${\mathrm{IHC}}^{+}≙1$, ${\mathrm{IHC}}^{-}≙0$ ) was subjected to logistic regression versus gene expression (*x*_Expr_). Responsibility functions for receptor positivity, *r*_+_, (dotted red curve) and *r*_−_ (dotted blue) were thus obtained. It will be shown later (Equation (4)) that *r*_+_ has to be multiplied by an upper limit, ${\hat{\alpha }}_{\mathrm{Expr}}$, to obtain the actual belief *α*_Expr_, see the dashed red curve. Likewise for *β*_Expr_ (dashed blue). Uncertainty: ochre. For a given expression value, e.g., *x*_Expr_ = 10, one can read off belief in positive (*α*), belief in negative (*β*) and uncertainty (*θ*). Note that analog concepts apply to any other gene of the full model.

**Figure A1:**
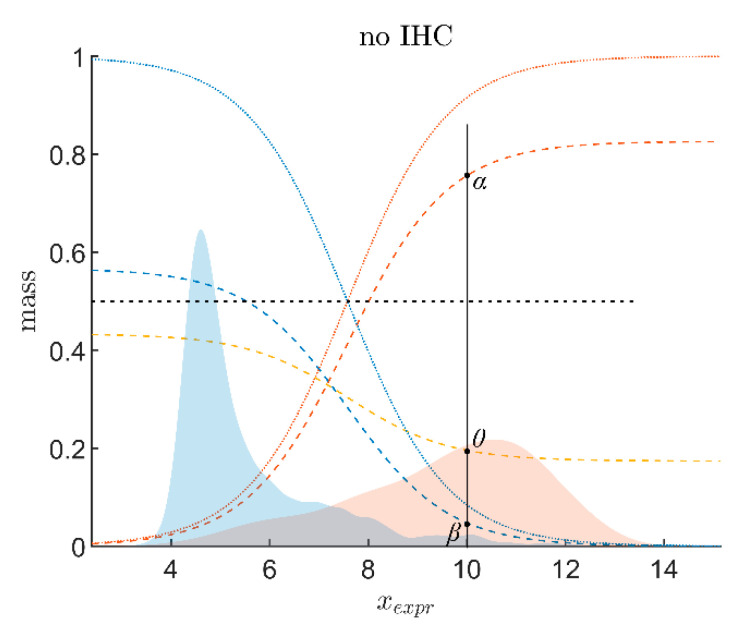
Logistic regression to obtain responsibility functions for progesterone, gene PGR. Distribution of gene expression for positive receptor (according to IHC) computed from density kernel estimates [71,72,73], shown red shaded, for negative IHC, blue shaded. Responsibility functions for receptor positivity, *r*_+_ (dotted red curve) and *r*_−_ (dotted blue) were obtained in this way. Belief in positive (*α*): solid red, belief in negative (*β*): solid blue and uncertainty (*θ*): solid beige.

Receptor status is related to gene expression (*x*_Expr_) as follows: The responsibility function for positive receptor status, $r_{+}$, defines the probability for a positive receptor status, given the expression value *x*_Expr_. Likewise, $r_{-}$ relates to negative receptor status. We used logistic regression

(1) $$\begin{array}{c} r_{+}\left(x_{\mathrm{Expr}}|c_{0},c_{1}\right)=\frac{\exp\left(c_{0}\;+\;c_{1}x_{\mathrm{Expr}}\right)}{1+\exp\left(c_{0}\;+\;c_{1}x_{\mathrm{Expr}}\right)}\\ r_{-}\left(x_{{}_{\mathrm{Expr}}}|c_{0},c_{1}\right)=1-r_{+}\left(x_{\mathrm{Expr}}|c_{0},c_{1}\right) \end{array}$$

and estimated the parameters $c_{0}$ and $c_{1}$ against IHC-measurements, separately for each gene and co-gene, for results see Table A2. Figure 2 shows the responsibility function *r*_+_ for positive estrogen (red dashed curve). *r*_−_, for negative estrogen (blue dashed curve), is based on the same regression coefficients, see Equation (1). A similar analysis was performed for progesterone, see Figure A1 for graphics and Table A2 for numerical values.

#### 2.2.2. Evidence of Receptor Status Based on Expression of Receptor Gene

Based on logistic regression, gene expression measurements lend themselves to derive evidence of receptor status according to Dempster–Shafer decision theory [70]. In the following, we formulate rules and principles in general terms of ‘gene expression’, *x*_Expr_, to keep notation general (later on, the first example with real data will specifically refer to *estrogen* receptor diagnostics. Even later, the very same procedure will be applied to progesterone).

Assume the variable gene expression, *x*_Expr_, is prognostic for receptor status. Given a measurement of *x*_Expr_, DST attributes *two* independent numbers, as outlined below:

- $\alpha_{\mathrm{Expr}}\left(x_{\mathrm{Expr}}\right)$: the belief (sometimes also called ‘degree of belief’ or ‘credibility’ [74]) for receptor status being positive on good grounds or by quality of the measuring method that has yielded *x*_Expr_;
- *β*_Expr_: the belief (probability) for receptor status being non-positive (i.e., negative) on good grounds or by quality of the measuring method;
- *θ*_Expr_ is a third quantity considered: the probability that the receptor status is uncertain.

*α*, *β* and *θ* are also called ‘masses’ of the respective outcomes. They are by definition larger than or equal to zero ($\alpha_{\mathrm{Expr}}\geq 0,\;\beta_{\mathrm{Expr}}\geq 0$, $\theta_{\mathrm{Expr}}\geq 0$), and if a mass equals zero, in our setting zero corresponds to the ‘empty set’, i.e., an outcome that will never be found [75]. Masses always add up to unity, and hence we talk about normalized mass functions [76]:

(2) $$\alpha_{\mathrm{Expr}}+\beta_{\mathrm{Expr}}+\theta_{\mathrm{Expr}}=1$$

Hence a third number is in fact redundant (may always be computed from the other two). Decision theory even considers a fourth quantity, called plausibility; it is also redundant but intuitive and useful:

(3) $$pl_{\mathrm{Expr}}=\alpha_{\mathrm{Expr}}+\theta_{\mathrm{Expr}}=1-\beta_{\mathrm{Expr}}$$

$pl_{\mathrm{Expr}}$ indicates the probability of a positive status being plausible, given the measurement *x*_Expr_ as is. The plausibility of a given outcome sums up everything either supportive or neutral, but excludes everything advocating the opposite outcome. The exactly opposite outcome is represented by *β*_Expr_.

The output of above procedure is the evidence $\left(\alpha_{\mathrm{Expr}},\beta_{\mathrm{Expr}}\right)$ for receptor status, based on the expression (*x*_Expr_,) of a gene (in general); data in Figure 2 were shown for the receptor gene of estrogen (ESR1). Note that finally, in the ‘full’ model, 4 such pieces of evidence (4 pairs of numbers) will be obtained: (1) for the estrogen receptor gene and (2) its co-gene; (3) for the progesterone receptor gene and (4) its co-gene.

The beliefs in receptor positive, *α*_Expr_, and negative, *β*_Expr_, may be obtained from gene expression alone, *x*_Expr_, as demonstrated above. Doing so, maximum expression corresponds to a responsibility function *r*_+_(*x*_Expr_) close to 1, see Figure 2. However, not even a gene expression that large can guarantee that the receptor is truly positive. Hence, the belief in positivity, *α*_Expr_, actually must be less than 1.

We chose to model this fact by a factor, $\hat{\alpha }$, called ‘upper limit for belief’ in Table A2. For details of calculation see Appendix A.4 and Appendix A.5. 

All in all we obtain the belief in receptor positivity after measuring $x_{\mathrm{Expr}}$:

(4) $$\alpha_{\mathrm{Expr}}\left(x_{\mathrm{Expr}}\right)={\hat{\alpha }}_{\mathrm{Expr}}\cdot r_{+}\left(x_{\mathrm{Expr}}|c_{0},c_{1}\right)\;\beta_{\mathrm{Expr}}\left(x_{\mathrm{Expr}}\right)={\hat{\beta }}_{\mathrm{Expr}}\cdot r_{-}\left(x_{\mathrm{Expr}}|c_{0},c_{1}\right)$$

*α*_Expr_ is represented by the increasing solid red curve in Figure 2, *β*_Expr_ by the declining blue one. The remaining uncertainty, *θ*_Expr_, is easily computed from reformulating Equation (2)

(5) $$\theta_{\mathrm{Expr}}\;=1-\alpha_{\mathrm{Expr}}-\beta_{\mathrm{Expr}}$$

and is shown as ochre curve in Figure 2. The two numbers (*α*_Expr_, *β*_Expr_) are collectively called ‘evidence’ of receptor status, given a measurement of the continuous variable ‘gene expression’, *x*_Expr_. They enrich the information given by a single number, the probability *p*, known from conventional statistics, quantifying the chances of receptor statuses, a similar procedure applies to the receptor gene of progesterone, see Figure A1.

#### 2.2.3. Combining Evidence from Receptor Gene Expression and IHC

To further increase precision of receptor status diagnostics, evidence from gene expression (*α*_Expr_, *β*_Expr_) and evidence from IHC (*α*_IHC_, *β*_IHC_) are combined by so-called ‘evidence combination rules’ (ECR). DST offers several such rules [69,74], out of which we consider two, the ‘Dempster–Shafer’ ECR, ⊕_D_, and the Yager ECR, ⊕_Y_ [77,78]. We chose the Yager rule, as it more easily accommodates contradicting items of evidence, see also Section 4.4 in the discussion. Performing some algebra, as detailed in our previous work [37], one finally obtains:

(6) $$\alpha_{\mathrm{Rez}}=\alpha_{\mathrm{Expr}}\alpha_{\mathrm{IHC}}+\theta_{\mathrm{Expr}}\alpha_{\mathrm{IHC}}+\alpha_{\mathrm{Expr}}\theta_{\mathrm{IHC}}\;\beta_{\mathrm{Rez}}=\beta_{\mathrm{Expr}}\beta_{\mathrm{IHC}}+\theta_{\mathrm{Expr}}\beta_{\mathrm{IHC}}+\beta_{\mathrm{Expr}}\theta_{\mathrm{IHC}}\;\theta_{\mathrm{Rez}}=\theta_{\mathrm{Expr}}\theta_{\mathrm{IHC}}+\alpha_{\mathrm{Expr}}\beta_{\mathrm{IHC}}+\beta_{\mathrm{Expr}}\alpha_{\mathrm{IHC}}$$

**Figure 3:**
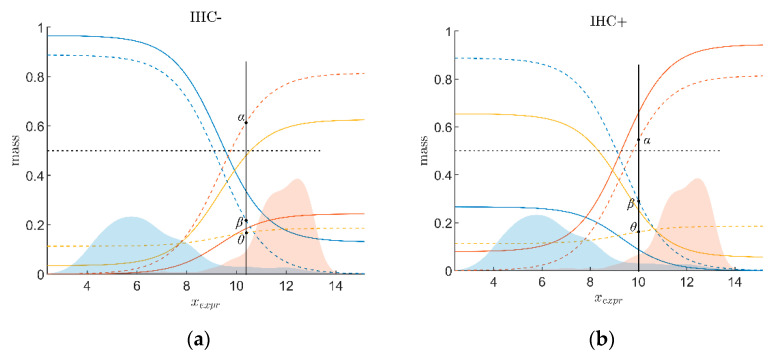
Combining receptor gene expression and IHC. Taking the estrogen receptor as an example, we demonstrate the principles of combining evidence from gene expression and IHC by the Yager evidence combination rule. (**a**) For IHC-negative estrogen receptor status. (**b**) For IHC-positive estrogen receptor status. Dotted lines represent beliefs merely based on gene expression, without considering IHC estimates (identical to the beliefs in Figure 2). Beliefs for gene expression combined with IHC estimates (via Yager evidence combination rule) are shown in solid lines. Clearly, a negative IHC estimate (**a**) strengthens the belief in negative (solid blue runs above dotted blue) and weakens the belief in positive (solid red runs below dotted red) for a given expression value, *x*_Expr_. As opposed, a positive IHC estimate (**b**) strengthens the belief in positive (red) and weakens the belief in negative (blue).

As IHC-evidence is made up of two sets of constants, combination with gene expression yields two sets of curves, one for IHC^−^ and one for IHC^+^, see Figure 3.

A definite decision for positive receptor status is obtained if the combined evidence exceeds 0.5 (*α* > 0.5). In that case the belief in positive surmounts the sum of both other beliefs (*β* + *θ* ≤ 0.5) and dominates. Hence, the dotted line *α* = 0.5 represents a decision border and will be analogously outlined in the following figures.

#### 2.2.4. Ternary Plots of Evidence for Personalized Medicine: A Primer

**Figure 4:**
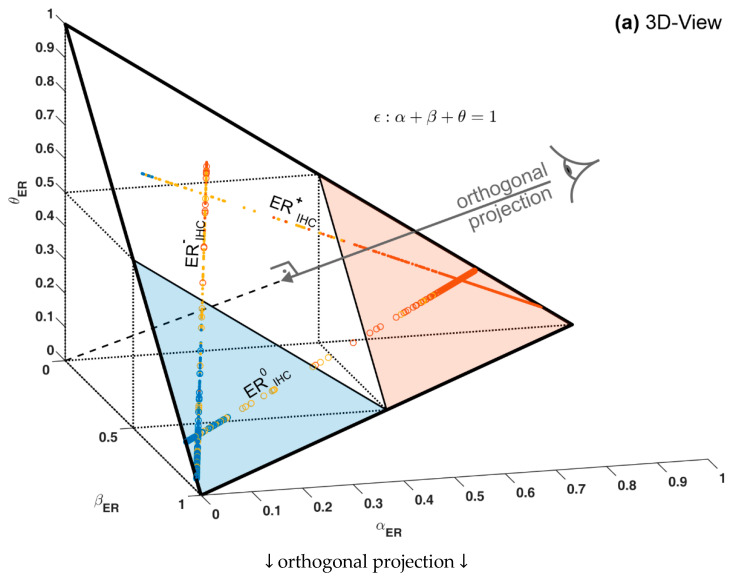

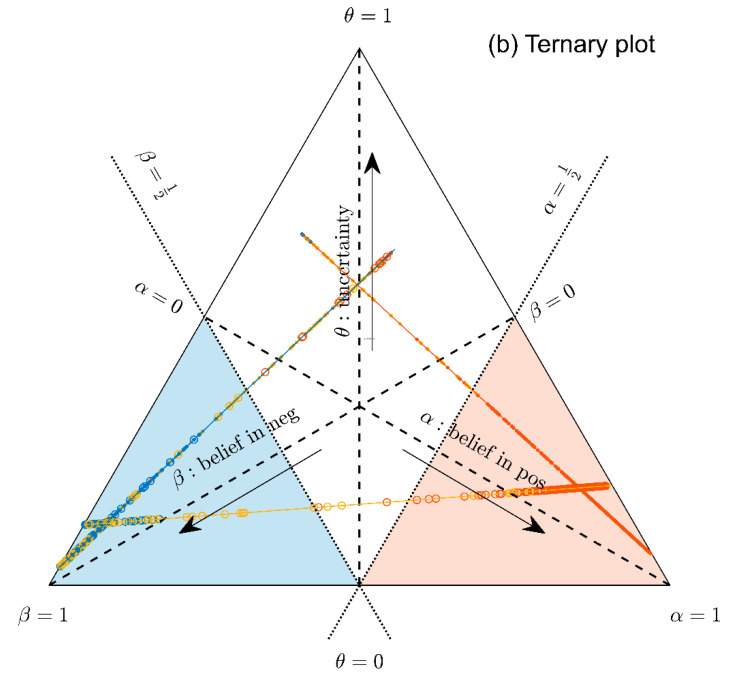
3D plot and ternary plot of evidence for estrogen receptor status. (**a**) ordinary 3D plot. (**b**) ternary plot. Orthogonal projection of the 3D scatter plot (as indicated) in panel (**a**) yields the ternary version, panel (**b**). In each corner, one piece of evidence dominates and both others are zero, e.g., (*α* = 1, *β* = 0, *θ* = 0 in the lower right corner). Note that the baseline of the ternary runs along the diagonal through the bottom plane of the 3D plot: along this bottom side, *α* runs from zero to 1 from left to right and *β* in reverse (right to left), hence sides of a ternary triangle do not represent usual ordinate axes, please refer to the tutorial (2.2.4). Midway points of triangle sides mark decision boundaries, e.g., *α* = *β* = 0.5 between positive and negative. Triangular areas contain definite results, either positive (*α* ≥ 0.5, shaded red) or negative ones (*β* ≥ 0.5, shaded blue). The kite-shaped area (white) represents undecided status according to DST. For each value of ER_IHC_ see labeling (${\mathrm{ER}}_{\mathrm{IHC}}^{+}$, ${\mathrm{ER}}_{\mathrm{IHC}}^{-}$, ${\mathrm{ER}}_{\mathrm{IHC}}^{u}$ ) evidence lies on a specific straight line due to mathematical reasons; samples with known IHC are shown as dots, samples with unknown IHC as circles. Coloring of samples *according to ODDS method, not DST*. Samples positive according to ODDS (red) may well lie within the undecided region according to IHC, etc.

Note that belief, plausibility and uncertainty are not independent but always sum up to unity for a given sample, see Equation (2). This mathematical property allows for a special graphic display, called ‘ternary plot’, as follows. When plotting these data in an ordinary 3-dimensional scatter plot with coordinates (*α*, *β*, *θ*), points of all samples lie within a single plane (of evidence), see Figure 4a. This is due to Equation (2), which—in mathematical terms—is nothing else than the equation of a plane in three dimensions [79]. This ‘plane of evidence’ may be viewed in orthogonal projection (https://en.wikipedia.org/wiki/Orthographic_projection (accessed on 26 March 2022)) which still contains all information but fits into two dimensions and is called ‘ternary plot’, see Figure 4b. 

Ternary plots are widely used in technology and science but have only marginally entered the medical sciences [80]. They might also gain importance in personalized medicine but deserve some skillful understanding. Hence we provide a short primer.

A ternary plot is powerful whenever three quantities (hence the name ‘ternary’) add up to a constant, for each individual considered. For example, a biological fluid (say milk) may be composed of water, protein and fat (three components) and nothing else. Clearly, the percentages of water, protein and fat must then add up to 100%. For a set of milk samples, these percentages may be visualized by points in a 3D scatter plot, such as Figure 4a. If we consider mixtures of different composition (e.g., skimmed, normal and fat milk and many other possible kinds) and plot their corresponding 3D points, we will be surprised to realize that all these points lie within a single flat plane in 3-dimensional space. The reason is a mathematical one: if coordinates always add up to a given constant, this is the very representation of a plane in mathematical terms [79]. This may be fruitfully exploited for personalized medicine as follows:

What is true for three components of a substance (milk) is also true for evidence composed of three numbers, *α*, *β* and *θ*, since they also add up to unity due to Equation (2). Hence, points of evidence (*α*, *β*, *θ*) for any single patient, lie within the same (2-dimensional) plane in 3D. This plane always lies in the same, specific position and orientation, for the following reason: the point (*α =* 1, *β =* 0, *θ =* 0), represents a valid point of evidence (adding up to unity) and must be part of the plane. Therefore, the plane cuts the *α*–axis at *α =* 1, see Figure 4a. Likewise, the plane also cuts both other axes at *β =* 1 and *θ =* 1, respectively. This uniquely defines an equilateral triangle in the 3D coordinate system, see Figure 4a.

Even though the plane of evidence lies embedded in a 3D coordinate system, it is by itself just a 2-dimensional object, as every flat plane is. Therefore, without any loss of information regarding the location of points (representing evidence) we may perform an orthogonal projection along the heavy arrow shown in Figure 4a. This yields a so-called ‘isometric view’. The triangle, viewed face-on, appears equilateral, now in two dimensions, see Figure 4b.

**Figure 5:**
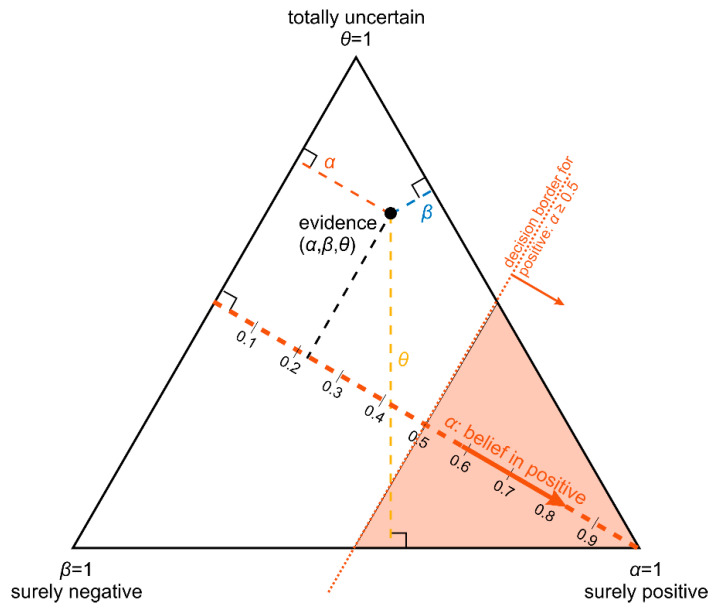
Principles of a ternary plot: obtaining coordinates by the altitude method. Decision border *α* ≥ 0.5: ‘positive to the best of our knowledge’ or ‘positive is more likely than anything else’.

A ternary plot does not have its axes at right angles, as ordinary plots do. To read off the coordinates of a point from such a ternary plot, several methods are available, out of which we propose the following (altitude method), illustrated in Figure 5:

Each of the three components of evidence, e.g., *α*, the ‘belief in positive’, has its own scale, see the dashed heavy line in orange; it starts on the left with *α* = 0 at a right angle from a triangle’s left side and runs towards the opposing corner, where *α* = 1 (indicating ‘surely positive’). See also the scale with numerical values aside. The two other scales, for *β* and *θ*, are defined analogously (not shown for simplicity).

Given some point within the ternary plot (see the heavy black dot in Figure 5), corresponding evidence components (*α*, *β*, *θ*) can be read off as follows. Note the lines being drawn perpendicular to each side of the triangle (light dashed lines in red, blue and beige)—they represent the axes for quantification. Values *α*, *β*, *θ* (red, blue, and beige, respectively) can be read off from the corresponding axis’ scale. In this example (Figure 5), the plotted point of the evidence produces a reading of *α* ≅ 0.22.

Note also the following intriguing features of this ternary plot:

- Parallel lines at right angles with one axis represent constant values for the respective variable (as with ordinary right-angle axes). In particular, the line crossing the *α*–axis at *α* = 0.5 (dotted red) discriminates points with *α* ≤ 0.5 (left upper) from those with *α* > 0.5 (towards lower right corner), and hence represents a decision border; points right of this border are predicted ‘positive’, since their evidence for positive is greater than for all other options (‘negative’ and ‘uncertain’) taken together.
- Decision borders segregate subsets of samples. For example, all samples within the triangle in the lower right of *α* = 0.5 (shaded light red) comprise samples predicted positive. Similarly, the subsets of negative and uncertain samples may be defined, see Figure 4b.
- In each corner one piece of evidence totally dominates, assuming a value of unity (*α* = 1: ‘surely positive’; *β* = 1: ‘surely negative’ and *θ* = 1: ‘totally uncertain’).
- Conversely, the footing point of each axis (e.g., *α* = 0) means that there is no indication whatsoever for the prediction at opposing corner. For example, *α* = 0 along the left side of the triangle, means that there is no indication whatsoever for a ‘positive’ prediction. All evidence is shared between ‘negative’ and ‘uncertain’ (*β* and *θ*). In this case *β* + *θ* = 1.
- A special role is played by the triangle’s bottom edge, running from *β* = 1 (left) towards *α* = 1 (right): for each sample along this line uncertainty *θ* equals zero, and all evidence is shared between belief in positive (*α*) and belief in negative (*β*), e.g., *α* = 0.6 and *β* = 0.4, while *θ* = 0. One may legitimately ask: “Does this mean that the prediction was made for sure?”. Since *α* > 0.5 and dominates both other options, we consider this prediction clearly positive. However, *α* = 0.6 is no more than a probability and not that much larger than the probability of the opposite outcome, *β* = 0.4. In reality, the outcome may well result in a negative prediction. If *θ* = 0, evidence masses revert back to ordinary probabilities: *p*^+^ = 0.6 for positive and hence *p*^−^ = 0.4 for negative, without indicating any uncertainty about the estimates of these two numbers. Thus, for *θ* = 0, decision theory’s evidence coincides with ordinary probabilities. In DST terminology the evidence is said to turn ‘Bayesian’ [74].
- In general, for *θ* > 0, decision theory not only gives estimates for probabilities (*α*, *β*) but additionally indicates the uncertainty of those (*θ*). It hence offers a wider scope of evidence, valuable in particular for personalized medicine.

Ternary plots allow for a highly transparent comparison of our two classification methods (ODDS versus DST) for each single sample:

- The *location of the point indicates the prediction according to DST* shown by the respective area: red triangular area for positive (+), blue for negative (-) and the white, kite-shaped area for inconclusive (inc).
- At the same time, *coloring of points indicates prediction according to ODDS*. For most samples, both predictions match. For some samples however, they differ, thus perfectly outlining the contrast between the two prediction methods.

Although ternary plots may seem somewhat unusual for medical application, they offer the unique capability to display *three* variables in *two* dimensions, provided their sum is constant, which is true for evidence and many other variables. We think it worth the effort to introduce ternary plots into the field of personalized medicine. They are the most adequate tool for quantitatively presenting evidence, and may in the future represent a cornerstone of personalized medicine.

### 2.3. Full Model: Evidence, Based on IHC, Genes, Co-Genes

In Section 2.2.1, Section 2.2.2, Section 2.2.3 and Section 2.2.4 and Appendix A.6 description was restricted to the receptor gene (no co-gene considered) in order to explain more transparent details. Now we revert to the whole model, including co-genes, see the flow chart of evidence in Figure 1. 

First, we supplement estrogen expression evidence $\left(\alpha_{\mathrm{Gen}},\beta_{\mathrm{Gen}}\right)$ by evidence $\left(\alpha_{\mathrm{Co}},\beta_{\mathrm{Co}}\right)$ from its co-gene, AGR3; the very same procedure outlined in Section 2.2.1 and Section 2.2.2 is carried out to obtain these results, see Table A2.

#### 2.3.1. Progesterone Evidence

Numerical results of the logistic regression for progesterone are shown in Table A2, for responsibility functions, see Figure A1. The co-gene of progesterone, incidentally, was estrogen, see Table A2.

#### 2.3.2. Combining Evidence Form Genes and Co-Genes

Next, evidence from genes and co-genes are combined by the Dempster Evidence Combination Rule (${⊕}_{D}$) to obtain the joint evidence from gene expression:

(7) $$\left(\alpha_{\mathrm{Expr}},\beta_{\mathrm{Expr}}\right)=\left(\alpha_{\mathrm{Gen}},\beta_{\mathrm{Gen}}\right){⊕}_{D}\left(\alpha_{\mathrm{Co}},\beta_{\mathrm{Co}}\right)$$

In detail, the Dempster rule [77] reads:

(8) $$\begin{array}{l} \alpha_{\mathrm{Expr}}=\frac{\alpha_{\mathrm{Gen}}\alpha_{\mathrm{Co}}\;+\;\theta_{\mathrm{Gen}}\alpha_{\mathrm{Co}}\;+\;\alpha_{\mathrm{Gen}}\theta_{\mathrm{Co}}}{1\;-\;\alpha_{\mathrm{Gen}}\beta_{\mathrm{Co}}\;-\;\beta_{\mathrm{Gen}}\alpha_{\mathrm{Co}}}\\ \begin{array}{l} \beta_{\mathrm{Expr}}=\frac{\beta_{\mathrm{Gen}}\beta_{\mathrm{Co}}\;+\;\theta_{\mathrm{Gen}}\beta_{\mathrm{Co}}\;+\;\beta_{\mathrm{Gen}}\theta_{\mathrm{Co}}}{1\;-\;\alpha_{\mathrm{Gen}}\beta_{\mathrm{Co}}\;-\;\beta_{\mathrm{Gen}}\alpha_{\mathrm{Co}}}\\ \theta_{\mathrm{Expr}}=1-\alpha_{\mathrm{Expr}}-\beta_{\mathrm{Expr}}=\\ \;\;\;\;\;\;\;\;=\frac{\theta_{\mathrm{Gen}}\theta_{\mathrm{Co}}}{1-\alpha_{\mathrm{Gen}}\beta_{\mathrm{Co}}-\beta_{\mathrm{Gen}}\alpha_{\mathrm{Co}}} \end{array} \end{array}$$

Combination of gene and co-gene is carried out along the same lines for estrogen and progesterone.

#### 2.3.3. Combining Evidence from Gene Expression and IHC

As outlined in Section 2.2.3 for single gene case, we now combine the full gene evidence for estrogen with its IHC counterpart according to the Yager rule, see Equation (6), to obtain (*α*_ER_, *β*_ER_, *θ*_ER_). The very same is done for progesterone, yielding (*α*_PGR_, *β*_PGR_, *θ*_PGR_).

#### 2.3.4. Combining Estrogen and Progesterone Receptor Status

**Figure 6:**
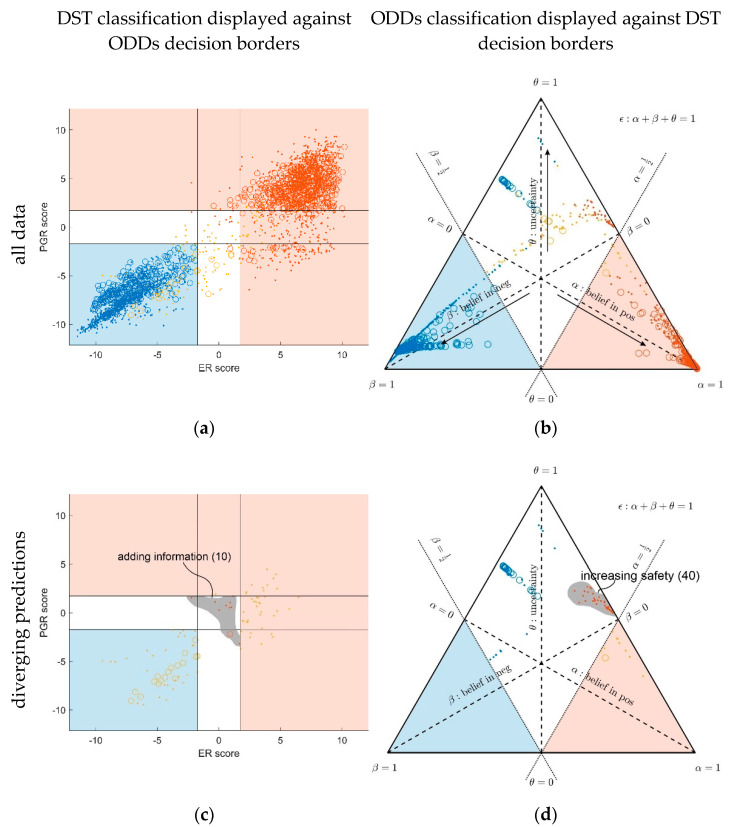
Hormone receptor status classified by ODDS versus Decision Theory. The same patient data were classified twice, along ODDS (left panels) and DST (right panels), for comparison. Data are shown as open circles if at least one IHC status is unknown. All other IHC statuses are shown as dots. Panels (**a**,**b**) include all 2519 patients of the cohort while panels (**c**,**d**) only display those 95 patients with predictions diverging between ODDS and DST, for easy comparison. Note the legends highlighting those patient samples which benefit from enhanced information or safety, respectively, conferred by DST. (**Left panels**): sample data arranged according to orthogonal ODDS score axes, but colored according to DST prediction. Light blue area: negative by ODDS. Light red area: positive. White L-shaped area: inconclusive by ODDS. (**Right panels**): sample data arranged according to ternary DST evidence axes, but colored according to ODDS prediction. Light blue triangle: negative by DST. Light red triangle: positive by DST. White kite-shaped area: inconclusive by DST. (**Lower panels**): only patients with diverging predictions are shown. Note two important groups marked by legends, corresponding to two cells in Table 1.

In the step to follow, evidence for different targets—estrogen and progesterone—will be combined. Clinically, a breast cancer patient is considered receptor positive, if either the estrogen ‘***OR***’ the progesterone receptor (or both) is/are positive, and treatment is assigned accordingly. Corresponding decision borders will be shown below (Figure 6). While clinical SOP (Standard Operating Procedure) draws on a crisp logical ‘***OR****’*, as implemented in the ODDS-method, DST offers a wider scope of possibilities. Evidence for estrogen (*α*_ESR_, *β*_ESR_, *θ*_ESR_) and progesterone (*α*_PGR_, *β*_PGR_, *θ*_PGR_) may be combined to obtain evidence for the overall hormone status (*α*_H_, *β*_H_) as follows [37]:

(9) $$\begin{array}{l} \alpha_{H}=\alpha_{\mathrm{ESR}}+\alpha_{\mathrm{PGR}}-\alpha_{\mathrm{ESR}}\alpha_{\mathrm{PGR}}\\ \beta_{H}=\beta_{\mathrm{ESR}}\cdot \beta_{\mathrm{PGR}} \end{array}$$

## 3. Results

### 3.1. Contrasting Predictions by ODDS versus DST

Predictions via conventional statistics (ODDS) and decision theory (DST) are directly compared for the whole patient cohort in Figure 6. To address clinical relevance, we highlight patients for which DST adds information (see legend), as well as those for which DST increases safety (see legend). For compactness, we abbreviate notation of the IHC receptor status, e.g., ${\mathrm{ER}}_{\mathrm{IHC}}^{-},{\mathrm{PGR}}_{\mathrm{IHC}}^{+}≙\left(-,+\right)$ or ${\mathrm{ER}}_{\mathrm{IHC}}^{+},{\mathrm{PGR}}_{\mathrm{IHC}}^{u}≙\left(+,0\right)$, with ‘0’ representing ‘undefined’. Likewise, we denote predictions (via ODDS or DST) as ‘neg‘, ‘pos’ and ‘inc’, with ‘inc’ representing ‘inconclusive’.

Note the following features in Figure 6:

- **In the left panels, samples are geometrically located according to ODDS scores, but color-coded according to DST prediction**.
- Decision borders in ODDS can be directly displayed in an orthogonal, 2-dimensional plot of ‘scores’, see Figure 6, left panels. Decision borders are defined by specific values for each receptor score (ER score, PGR score), see our previous paper [37], and, hence, appear as vertical lines for estrogen and as horizontal lines for progesterone, respectively. The rectangular region (in faint blue) denotes receptor status predicted definitely negative, the L-shaped stripe (no color) denotes inconclusive status, and the L-shaped stripe (in faint red) definitely positive predictions.
- ODDS scores incorporate IHC evidence in an additive fashion. Each of the nine possible IHC statuses (+ +, − −, + −, − +, + 0, 0 +, − 0, 0 −, 0 0) merely differ in shifts along the respective ODDS coordinate (ER score, PGR score). ODDS decision borders are, hence, valid for any combination of IHC statuses.
- **In the right panels, samples are geometrically located according to DST evidence, but color-coded according to ODDS**.
- Decision borders in DST are most appropriately displayed in ternary plots of evidence, see Figure 6, right panels. Decision borders run along evidence *α* = 0.5 and *β* = 0.5, respectively, which appear as straight lines in a ternary plot. DST evidence also incorporates IHC information, and decision lines, hence, also represent unique borders in the ternary plot, valid for any combination of IHC statuses (+ +, − −, + −, − +, + 0, 0 +, etc.).
- **In the ternary plot, DST evidence for subsets of patient samples appear in polygonal areas**. In fact, these areas root in respective combinations of IHC statuses for estrogen and progesterone (+ +, − −, + −, etc.), as will be scrutinized in the appendix, for those interested in mathematical details. Indeed, these polygonal areas are generalizations of those simple straight lines already seen with single gene expression data (Figure 4). Since each receptor may assume three values (+, −, 0), there are 3^2^ = 9 possible IHC status combinations for two receptors. Some IHC statuses give rise to very distinct arrangements of samples, such as ‘lines’. Other IHC combinations give rise to more polygonal-shaped areas. Details will be discussed below. Data samples along these lines or polygons are seen to cross DST decision borders (dashed lines at *α* = 0.5 and *β* = 0.5, respectively). For example, if such a subset of samples crosses from inconclusive to decided, this indicates that IHC on its own was inconclusive, but adding evidence from (increasing) gene expression finally rendered a decision:
  ○ A stripe of red points originates within the DST-inconclusive, kite-shaped area and protrudes into the positive triangle.
  ○ The stripe of blue points originates in the DST-inconclusive, kite-shaped area and protrudes into the negative triangle.

Crossing decision borders for given IHCs underpins the importance of information from gene expression being added.

### 3.2. Clinical Relevance of DST versus ODDS

Agreement and divergence between ODDS and DST are summarized in Table 1. Note that both methods never definitely contradict each another (positive versus negative predictions for a given sample); see the zero counts in the corners off diagonal. Differences only occur for samples predicted as inconclusive. In 59 cases, both methods agree in yielding ‘inconclusive’. However, DST reports almost equal numbers of samples from ODDS-negative (45) and ODDS-positive (40) as DST-inconclusive, ending up with 144 inconclusive samples. Conversely, ODDS declares none from DST negative and only 10 from DST-positive as ODDS-inconclusive, ending up with just 69 samples rendered as inconclusive. In general, agreement between ODDS and DST is fine, with 999 + 59 + 1366 = 2424 out of 2519 samples (96.2%), as reflected by the high inter-rater agreement coefficient, Cohen’s kappa: *κ* = 0.9287 [81].

Besides good overall agreement, possible advantages of DST may be seen twofold, cf. the cells outlined with bold face in Table 1. The very same groups of patients are highlighted with legends in Figure 6:

- For 10 patients, DST predicted a positive receptor status, whereas ODDS had predicted ‘undecided’. Based on the additional information provided by DST, these patients may, upon careful reassessment, be candidates for milder therapies, possibly without chemotherapy (chemo). We, therefore, labelled this group with ‘adding information’ in Figure 6, panel (c).
- For 40 patients, DST predicted ‘undecided’, whereas ODDS had predicted ‘positive’. ‘Undecided’ severely questions abstaining from chemo and calls for a re-assessment at least. We, therefore, labelled this group with ‘increasing safety’ in Figure 6, panel (d).

**Figure 7:**
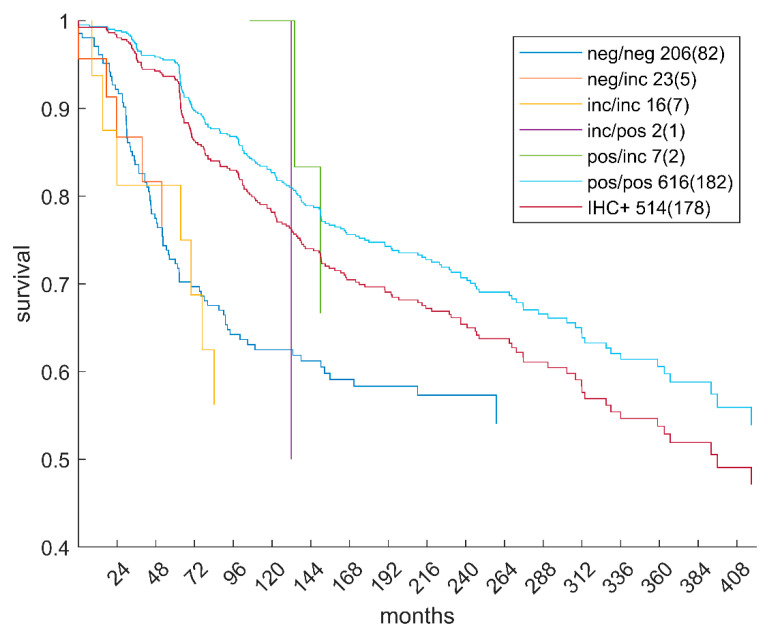
Survival free from recurrence. Kaplan–Meier estimates were obtained for several patient subgroups in Table 1. Legend acronyms refer to cells in Table 1 as ODDS/DST, figures give numbers of patients with survival data available and number of events (i.e., recurrences) in parenthesis. The curve ‘IHC+’ refers to patients diagnosed receptor positive according to current clinical standards, i.e., positive for estrogen or progesterone (or both).

Hormone receptor diagnostics—in comparison with ODDS and DST—was evaluated regarding its impact on survival. Figure 7 shows survival, free from recurrence, for several relevant subgroups listed in Table 1. Acronyms in the legend of Figure 7 correspond to those in Table 1, and figures in the legend give the numbers of patients with survival data available and number of events (i.e., recurrences) in parenthesis. Naturally, the two largest groups are those that ODDS and DST found in agreement (neg/neg, pos/pos)—they exhibit rich survival curves, with many patients and numerous events. Subgroups with disagreement between ODDS and DST (fortunately) contain only few patients, reflecting the fact that, already, ODDS was an advanced, accurate prediction method. The point of largest possible merit is the subgroup pos/inc: 40 patients considered positive by ODDS could have been deprived of chemotherapy, although being eventually negative. Within this group, survival data were available only for seven, relegating statistical testing meaningless.

For comparison, the IHC+ group was also evaluated, incorporating patients receptor positive either for estrogen *OR* progesterone, see Figure 7. Such patients are considered receptor positive and treated accordingly by ‘conventional’ clinical therapy allocation. Compared to these, our pos/pos group enjoyed definitely superior survival (log-rank *p* = 0.03). Since all patients considered in our study were actually treated according to conventional, clinical ‘IHC+’, we might speculate as follows: this actual, former treatment as ‘IHC+’ was confirmed post hoc in our study (by pos/pos) as correct and, hence, these patients experienced much better survival.

Over the years, hormone receptor status has become the most important predictive parameter, which allows for an identification of endocrine-sensitive invasive tumors. The use of hormone-receptor-targeted treatment strategies is associated with an approximately 50% reduction in recurrences and a reduction in breast-cancer-attributed deaths by approximately 30%, and receptor status assessment has, therefore, become the single most important biomarker in early and advanced breast cancer. A correct classification of endocrine sensitivity by receptor measurement is, therefore, critical for individualized treatment, since falsepositive results lead to overtreatment and therapy-associated side effects, which range from menopausal symptoms, infertility and depression, to bone loss and an increase in fractures, and other significant side effects. False negative results, on the other side, subject patients to under-treatment and a profound worsening of the long-term outcome. These profound clinical consequences are contrasted by a number of technical uncertainties: the hormone receptor status is presently assessed by immunohistochemistry, and different standards in tissue fixation, varying protocols, the myriad of commercially available antibodies, inter-observer variability and other technical issues compromise an objective assessment. Moreover, while some labs use a cut-off of 10% of hormone receptor positive cells, others prefer a cutoff of 1%, thus, limiting the value of the current gold standard in receptor assessment. Within this context, prediction models, such as DST and ODDS, can add to further ascertainment of the receptor status. The decision of which model to use could be factored into the decision tree and allow for a more personalized treatment, in the sense that the more conservative DST could be applied in older and frail patients, in whom the significant side effects of endocrine therapy need to be balanced against competing mortalities and might lead to an omission of endocrine therapy, and an additional IHC, performed by an independent laboratory could be helpful in decision making and in potentially sparing patients from therapy-associated side effects. By contrast, ODDS with 0.4% inconclusive rates might be more appropriate in mainstream assessment, since the need for independent reassessment can be reduced.

### 3.3. Specific Differences in Prediction between ODDS and DST

As noted above, definite predictions were never seen contradicting between ODDS and DST. However, decisions deemed definite in ODDS were rendered inconclusive by DST and vice versa. This becomes evident by contrasting predictions *coded by location* versus predictions *coded by color* in Figure 6:

- Within the plane of ODDS scores (left panel), the L-shaped area (colored faint red) denotes samples definitely predicted positive by ODDS (according to location). However, some of them are inconclusive according to DST (colored beige); in fact, 40 DST-inconclusive samples invade the positive, and the other 45, the negative domain of ODDS scores, see Table 1.
- Conversely, the uncolored L-shaped area accommodates samples predicted inconclusive according to ODDS (according to location). However, 10 are colored red, i.e., according to DST, decided positive. In fact, these samples, definitely predicted positive by DST, invade the inconclusive region of ODDS scores and are labelled ‘adding information’, see Figure 6, panel (c) and Table 1.
- Within the ternary plot of DST evidence (right panel), the triangular shaped areas denote samples predicted negative (faint blue) and positive (faint red), respectively, according to DST (by location). However, some samples are color-coded beige, i.e., they were rendered inconclusive by ODDS. Note that the very same samples appear in dual roles along ODDS scores and ternary evidence, respectively (left and right panel).
- Conversely, the uncolored kite-shaped area denotes samples predicted inconclusive according to DST (by location). However, some of them are color-coded red or blue, i.e., definitely predicted as positive or negative according to ODDS. In fact, 40 samples definitely classified positive through ODDS intrude into the ‘inconclusive’ region of DST and have been labelled as ‘increasing safety’, see panel (d). Another 45 definitely predicted negative through ODDS intrude into the ‘inconclusive’ region of DST.

All in all, differences in prediction only occur with samples on the brink of predictability. While one method yields positive or negative, the other may yield ‘inconclusive’. These differences turn up in the off-diagonal elements of Table 1, which are small; see also the percentages. Even if differences are small, they are important for the single patient and seen at the core of personalized medicine.

Moreover, visual inspection of the ternary plot in Figure 6 reveals samples not being evenly distributed over the triangular plane of evidence. Samples, rather, appear in groups, arranged in lines or lengthy polygons. The mechanisms behind the scenes, giving rise to these effects, are scrutinized in Appendix A.6 and Appendix A.7.

## 4. Discussion

Dempster–Shafer Decision Theory (DST) has been made available for the personalized therapy of breast cancer in a previous paper [37], in particular, to increase the precision of receptor status assessment. Unfortunately, we could not map with ground truth in our papers, since ground truth is not available for the data used. However, we were able to provide a sound comparison between ODDS and DST and pinpoint particular differences in performance. To underpin the usefulness of DST, we have scrutinized the survival of patients with status corrected from positive or negative predictions by ODDS towards ‘inconclusive’ by DST, see Figure 7. Since only a small fraction of patients was to be ‘corrected’ (see Table 1), survival curves degenerate and were included only for completeness. Even if this percentage is small, it seems mandatory, considering the large number of breast cancer patients. In practice, patents rendered inconclusive should receive lab reassessment, in order to reduce false estimates and increase precision. 

In addition, we compared patients considered receptor positive according to up-to-date clinical standards (IHC+, red curve) with those considered positive (pos/pos, light blue curve) according to both of our proposed methods, ODDS and DST. Patients positive according to the new methods experienced significantly better survival (log-rank *p* = 0.03) than those conventionally diagnosed positive, see the red versus the light blue curves in Figure 7.

Comparing ODDS and DST, DST was found to be somewhat more conservative than ODDS. Vice versa, patients considered ‘positive’ by DST, while being considered ‘undecided’ by ODDS, may benefit from this additional information inferred by DST. However, this gain of information has two sides: the ‘positive’ prediction might not really hold in the end, and relying on it may cause harm. Hence, re-evaluation remains the only safe advice in these cases.

### 4.1. Advantages of Evidence Compared to Probabilities in Conventional Statistics

In addition to our previous work, the implementation of DST is, here, unfolded in three steps:

First, we demonstrate the simplest case, starting with a single gene (the receptor gene) and demonstrate how to:

- Obtain DST evidence from gene expression.
- Obtain DST evidence from IHC.
- Fuse both items of evidence above, via the Yager evidence combination rule [78].
- Display results in a ‘ternary’ plot, a genuine format for presenting evidence.
- Show subgroups of patients with given IHC status, giving rise to specific patterns of samples in evidence space.

In a second step, we demonstrate how to create evidence from co-genes and join them with evidence of receptor genes and IHC (by Dempster and Yager Evidence Combination rule, respectively).

In the third step we demonstrate how to join evidence from estrogen with that from progesterone, using a formula imitating the clinical criterion ‘positive ER or positive PGR’ for ‘receptor positivity’, in terms of Dempster–Shafer mathematics.

This stepwise approach allows for a detailed introduction into ternary plots, demonstrating their applicability to clinical decision making, based on evidence. It becomes clear that evidence not only provides more information about the outcome of a measurement than conventional probability does, but that probabilities are supplemented by uncertainty. Evidence also has the property of three numbers summing up to unity for each single sample considered, and may be advantageously displayed in ternaries. Groups of patients (different IHC statuses) are segregated by the method itself (being either positive or negative).

Data quality is a crucial aspect of personalized medicine. In this work, we have never let gene expression overrule IHC. Technically, this was achieved by selecting the constants $\hat{\alpha }$ in our model very conservatively. As a consequence, positive IHC estimates were never converted into negative, not even a positive progesterone when estrogen was negative IHC = (−, +). Such IHC estimates occurred in 15 samples, and gene expression by itself would turn them into (−, −), if we had modeled less weight into IHC and more into gene expression.

### 4.2. How Uncertainty May Help Increase Correctness (Precision)

**Figure 8:**
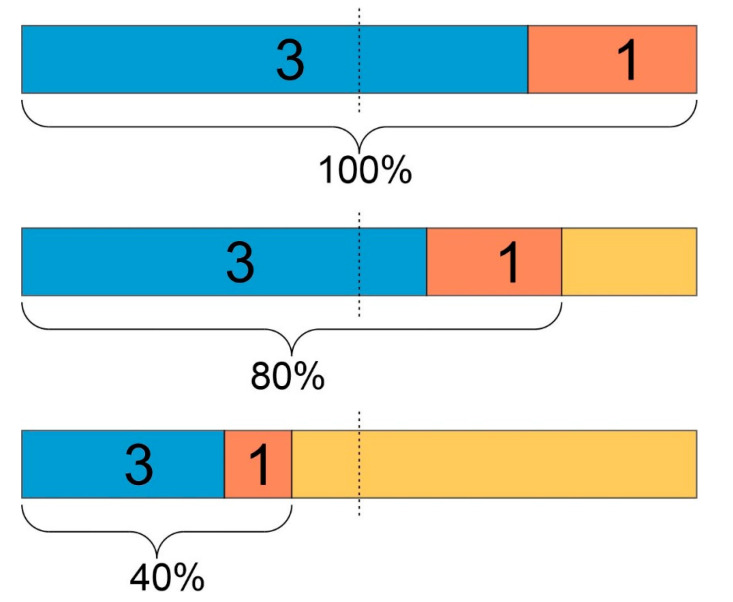
Uncertainty puts probabilities into perspective. Smaller voter turnout (e.g., 80%, 40%) in elections compares to increased uncertainty in DST. Dashed lines indicate 50%. From the very same ratio of votes for pro and contra (3:1 in each scenario), different consequences may be drawn in the light of high or low voter turnout, respectively. Likewise, probabilities of diagnoses may only be considered reliable if uncertainty, according to DST, is below some threshold.

At first glance, this statement may seem paradoxical. However, DST—in comparison to ODDS—supports this concept, as can be seen from a vivid comparison:

Suppose we have a ballot between two options (pro, contra). If the voter turnout was 100%, we might obtain 75% for pro and 25% for contra (3:1), and with full right, consider this a clear decision. The option ‘pro’ would clearly be implemented, having the majority of voters on its side, see the top bar in Figure 8. Exactly this scenario corresponds to classical statistics, considering a probability *p* and the probability 1 − *p* for its opposite.

Now suppose the voter turnout was only 80%, with exactly the same distribution between pro and contra, i.e., 60%:20% = 3:1, see the second bar in Figure 8. In this case also, we would consider it a valid decision, despite 20% non-voters, representing what is termed ‘uncertainty’ in DST. However, the result would not be considered as ‘robust’ as in the first case.

Finally, suppose a voter turnout of just 40%, again with the same ratio between pro and contra of 30%:10% = 3:1, see the third bar in Figure 8. Such a result would not be considered sound enough to draw conclusions from. An uncertainty of 60%—in terms of DST—may render the result ‘un-trustable’, even with a large ratio of probabilities p:(1−p)=3:1. After all, the relative ‘majority’ of 30% is far from absolute (50%). In such a case, a wise politician would not be confident to implement option ‘pro’, since opposition might emerge that is too strong to overcome.

Analog concepts hold for medical diagnostics. As DST introduces uncertainty as the third part of evidence [82,83], borderline or questionable results obtained by classical statistics may be relegated ‘uncertain’, suggesting further assessment and, thereby, increasing final correctness. In addition, significantly different risks may be inferred by falsepositive as compared to falsenegative decisions. For example, a falsepositive receptor status may lead to the avoidance of chemotherapy, in this case, the lifesaving therapy. Accordingly, one might request very low uncertainty, in order to ‘take a positive status serious’, regarding therapeutic consequences. Conversely, a false-negative status might ‘just’ entail unnecessary chemo, a comparatively lower risk. All in all, it is but a clinical decision how much uncertainty seems acceptable.

To allow for evidence-based decisions, the explicit quantification of uncertainty seems utmost desirable.

### 4.3. Extensions of Decision Rules

The approach presented here may be expanded by considering more than one co-gene, since DST allows us to combine more than two items of evidence. Considerable increases in stability can be expected if such expanded markers are applied to new incoming data.

Another possible extension refers to combination rules.

One basic concept for combining evidence from different sources was introduced by Dubois [84], hence, termed “evidence combination rule (ECR) after Dubois and Prade”. In the case of just two outcomes, this boils down to the Yager rule [78]. Smarandache [85] further generalized combination rules and defined the PCR5 combination rule, relevant for three (or more) outcomes. Fontani [66] proposed fusing the spaces of events in image processing and Denœux introduced weighted combination [69,74,75]. Chen defined distances between evidence [86]. Yang reviewed a framework of evidence combination rules and evidence weighting and discounting [65] and Sentz compiled all rules, in a comprehensive overview [87].

In the present work, we only used the Dempster Evidence Combination Rule (ECR) and the Yager rule [78]. However, this is not mandatory. In fact, a variety of ECRs exist, which differ in behavior in certain situations.

### 4.4. Modelling Sharp and Soft Clinical Decisions

The Dempster Evidence Combination rule advocates fierce decisions—leaving little uncertainty in the conclusions—even if both pieces of input evidence concede considerable uncertainty. As opposed, given the same input evidence, the Yager rule follows a much softer strategy, transmitting larger uncertainty into its conclusion. We illustrate this by a specific example.

Suppose we have two items of evidence for receptor status: The first piece of evidence, from gene expression (*α* = 0.8, *β* = 0.1, *θ* = 0.1), strongly favors ‘positive’, via large *α* and small *β*. Moreover, it claims to be quite ‘sure’ in terms of small *θ*. The second piece of evidence, from IHC (*α* = 0, *β* = 0.7, *θ* = 0.3), favors ‘negative’, with some larger uncertainty *θ* = 0.3. Obviously, these pieces of evidence contradict each other quite strongly, and one may legitimately ask ‘what should be the synthesis of these two?’ The answer can be precisely modelled by decision combination rules, according to Dempster (⊕_D_) or Yager (⊕_Y_), which also exemplifies their difference in approach.

For the current example, the Dempster rule (Equations (7) and (8)) yields as combined evidence *E*_D_ = (*α* = 0.54, *β* = 0.38, *θ* = 0.068), expressing a quite ‘sharp’ contradiction (large *α*, large *β*), without admitting much uncertainty (small *θ*). On the contrary, the Yager rule, Equation (6), yields combined evidence *E*_Y_ = (*α* = 0.24, *β* = 0.17, *θ* = 0.59), expressing only ‘soft’ contradiction (small *α*, small *β*), with quite a lot of uncertainty (large *θ*).

How can these features be exploited for personalized medicine?

Clinical experts have always been looking for the most beneficial balance in decision making, based on SOPs, their personal experience, and also skill, or even educated guessing, in particularly difficult cases. It has always been the strength and fame of top clinicians to decide correctly in a percentage of cases far above average. However, it may not be fully transparent how such an outstanding clinical performance comes about and could be transferred to young doctors in training. Decision theory tries to bring such ‘clinical expert competence’ down to more formally applicable rules. Of course, it will remain the task of top clinicians to help define and select those rules, based on sound statistical evaluations of clinical studies. Such decision rules, once established, may be incorporated in SOPs and will improve their performance significantly.

While this work exemplifies the use of DST in personalized medicine, related to the very specific field of breast cancer receptor diagnostics, the methods described are universal. Decision theory, in particular, the fusion of diverging evidence (sometimes also called ‘sensor-fusion’), as well as the professional incorporation of uncertainty into biomarker research, seem valuable for all fields of personalized medicine and medicine in general.

## Figures and Tables

**Table 1:** Prediction by DST (Dempster–Shafer decision Theory) versus ODDS (conventional statistics). Predictions negative (neg), inconclusive (inc) and positive (pos). No samples are classified as fully contradicting (positive versus negative). Differences arise from samples predicted ‘inconclusive’: 144 (5.7%) via DST compared to 69 (2.7%) via ODDS. DST predicts more conservatively than ODDS. Cells with numbers in bold represent patients with ‘gain of information (10)’ and ‘gain of safety (40)’, respectively. As an overall measure of agreement we computed Cohen’s kappa: *κ* = 0.9287 [81].

Number of Samples		DST			
		neg	inc	pos	sum
ODDS	neg	999	45	0	1044
	inc	0	59	**10**	69
	pos	0	**40**	1366	1406
	sum	999	144	1376	2519
**percentage**		**DST**			
		**neg**	**inc**	**pos**	**sum**
ODDS	neg	39.7%	1.8%	0.0%	41.5%
	inc	0.0%	2.3%	**0.4%**	2.7%
	pos	0.0%	**1.6%**	54.2%	55.8%
	sum	39.7%	5.7%	54.6%	100.0%

**Figure A2:**
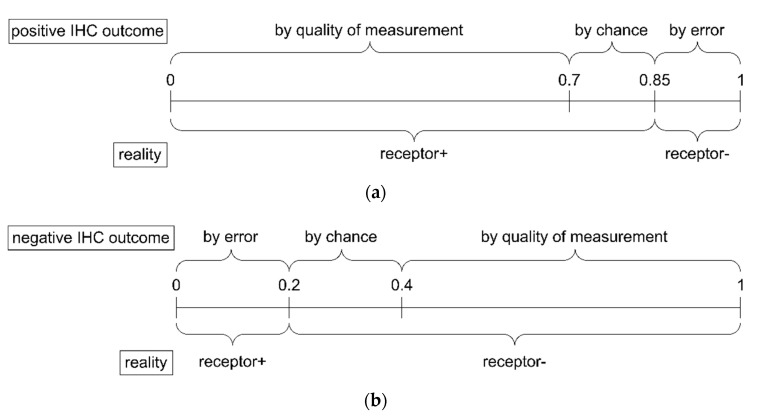
Results of measurements versus reality, seen along the concepts of Dempter–Shafer Theory. In panel (**a**) we focus on positive results only, yielded by IHC measurements (positive results represent 100%), see upper labels. Out of these, 70% are true positives (see lower labels) and result due to quality of the measuring technique. Accordingly, the belief in positive *α* = 0.7. Another 15% have come out as true positives by chance. The remaining 15% of positive outcomes are due to error, i.e., they are false positives, being truly negative. Both together represent the uncertainty ($\theta_{{\mathrm{IHC}}^{+}}^{}=0.3$) of being receptor positive in reality. In panel (**b**) we focus on negative measurement outcomes only: 60% of these come about due to the quality of measurement, 20% were correctly negative by chance and another 20% false negatives, since they are positive in reality.

**Figure A3:**
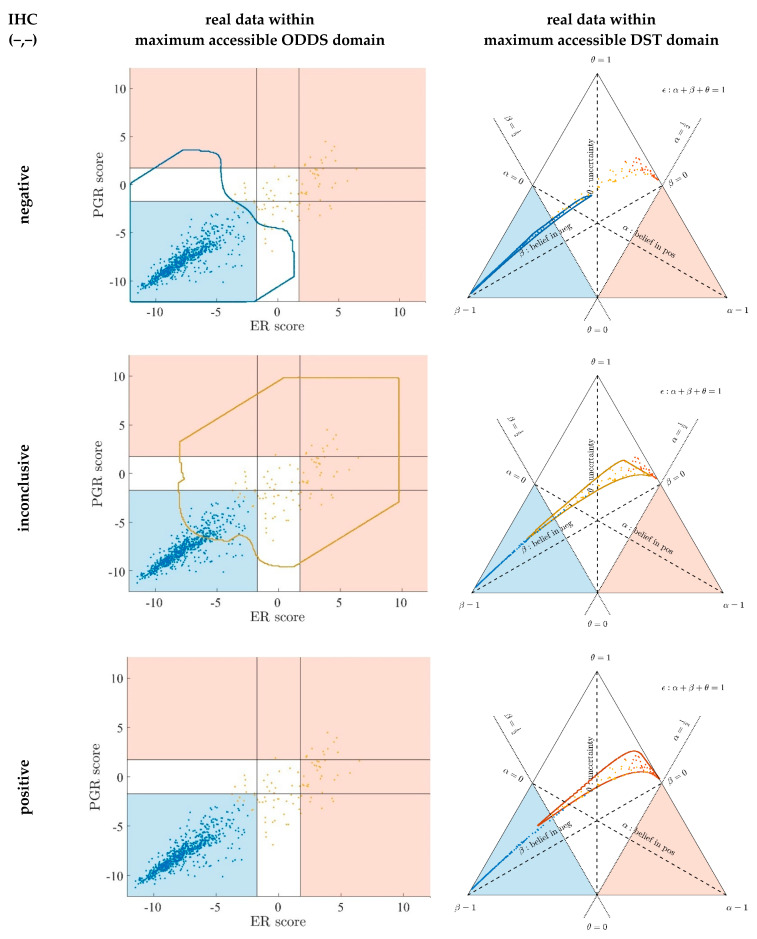
Real sample data within maximum accessible domains for IHC = (−,−). **Left column:** Real data shown in coordinates of ODDS prediction scores. Square in light blue (bottom left): criterion for negative predictions according to ODDS. Area in light red: criterion for positive predictions according to ODDS. In between (white): area of inconclusive ODDS predictions. Outlined areas represent maximum domains accessible for DST predictions, displayed in ODDS coordinates. Sample data, *located* according to their ODDS prediction scores but *colored* according to DST prediction (neg ≙ blue, inc ≙ beige, pos≙ red). **Right column:** Real data shown in ternary coordinates of DST prediction evidence. Triangle in light blue (bottom left): criterion for negative predictions according to DST. Triangle in light red (bottom right): criterion for positive predictions according to DST. White, kite-shaped area: criterion for inconclusive DST predictions. Outlined areas represent maximum domains accessible for ODDS prediction, displayed in DST coordinates. Sample data, while being located according to DST evidence is colored, however, according to ODDS prediction. **Rows 1–3**: Negative, inconclusive and positive predictions according to DST (left column) and ODDS (right column), respectively. **Purpose:** Differences between ODDS and DST predictions can easily be traced for real values as well as for maximum domains, e.g.: (1) Samples predicted negative by ODDS penetrate into the ‘inconclusive’ area (white kite) of DST (row 1, right panel). (2) The maximum domain for negative evidence by DST penetrates into the inconclusive (white) and also into the positive (light red) area of ODDS (row 1, left panel). For more extensive examples and extensive discussion, see text.

**Figure A4:**
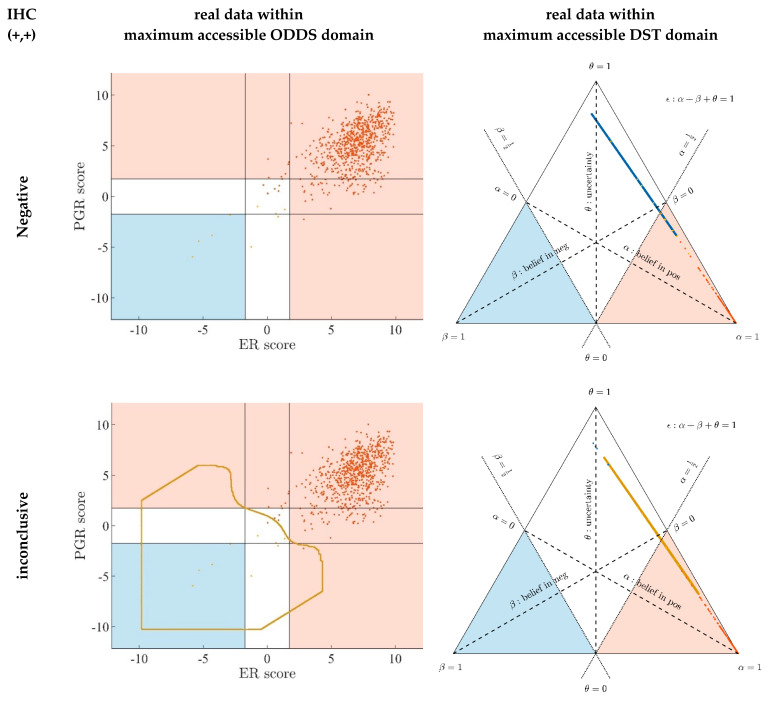

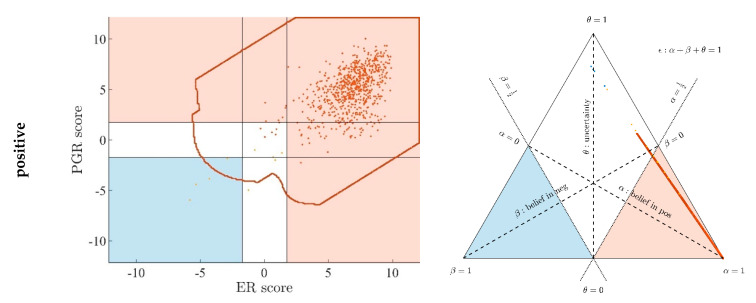
Real sample data within maximum accessible domains for IHC = (+,+). For general features of display see caption to Figure A3.

**Figure A5:**
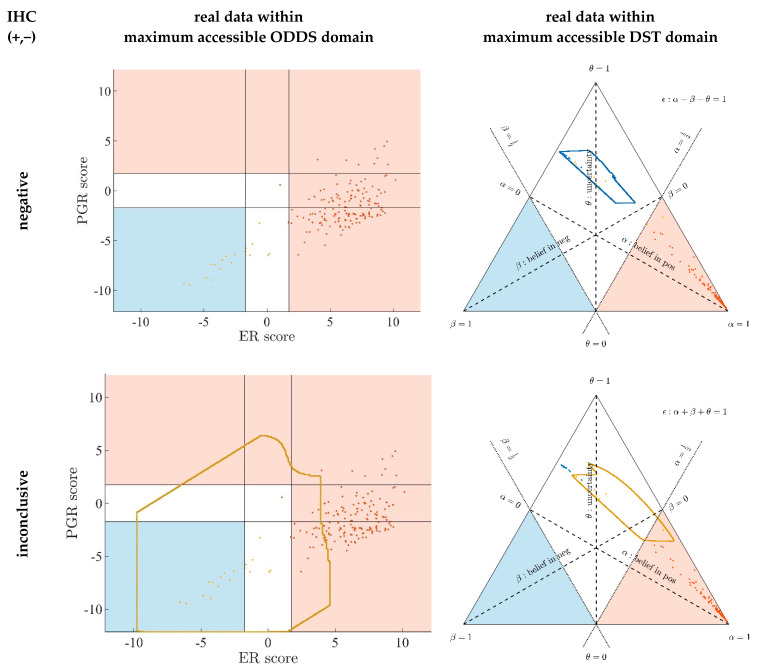

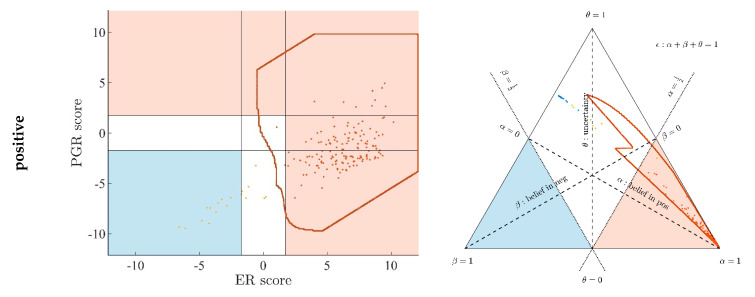
Real sample data within maximum accessible domains for IHC = (+,-). For general features of display see caption to Figure A3.

**Figure A6:**
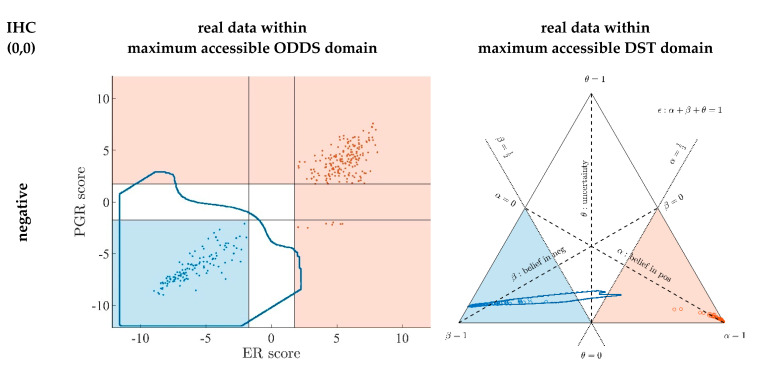

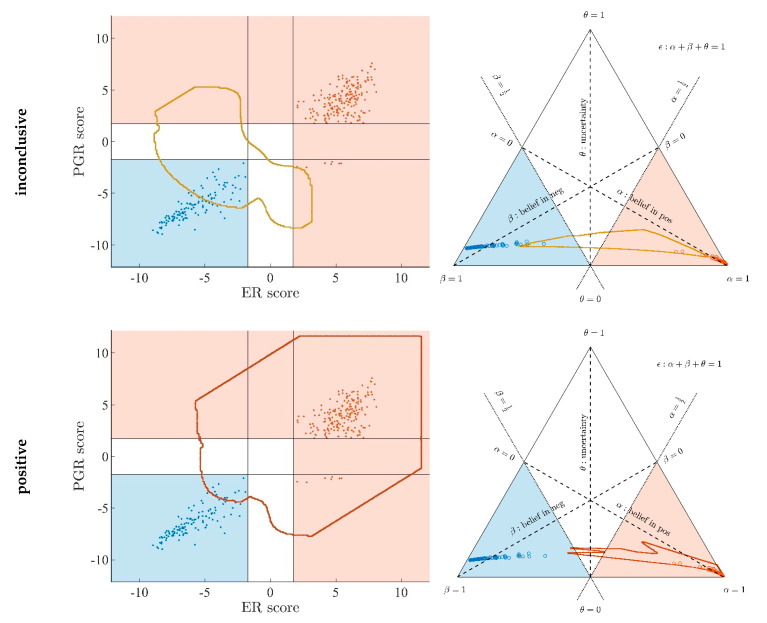
Real sample data within maximum accessible domains for totally unknown IHC = (0,0). For general features of display see caption to Figure A3.

**Figure A7:**
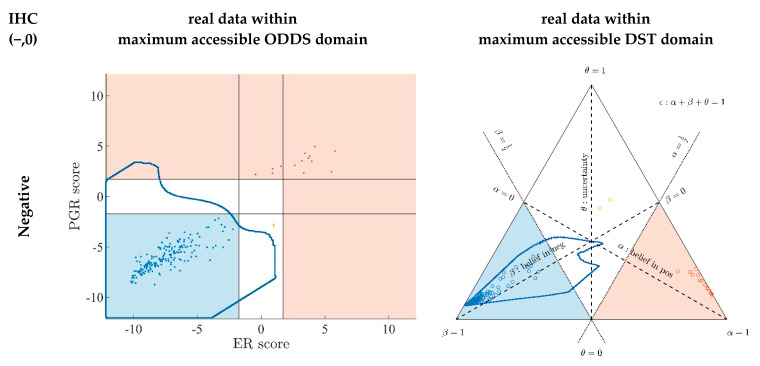

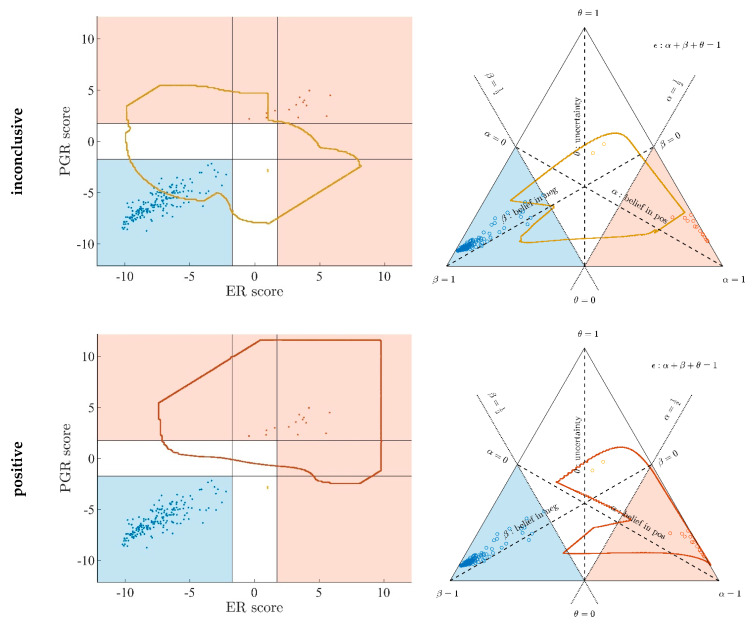
Real sample data within maximum accessible domains for the IHC case = (−,0). For general features of display see caption to Figure A3.

**Figure A8:**
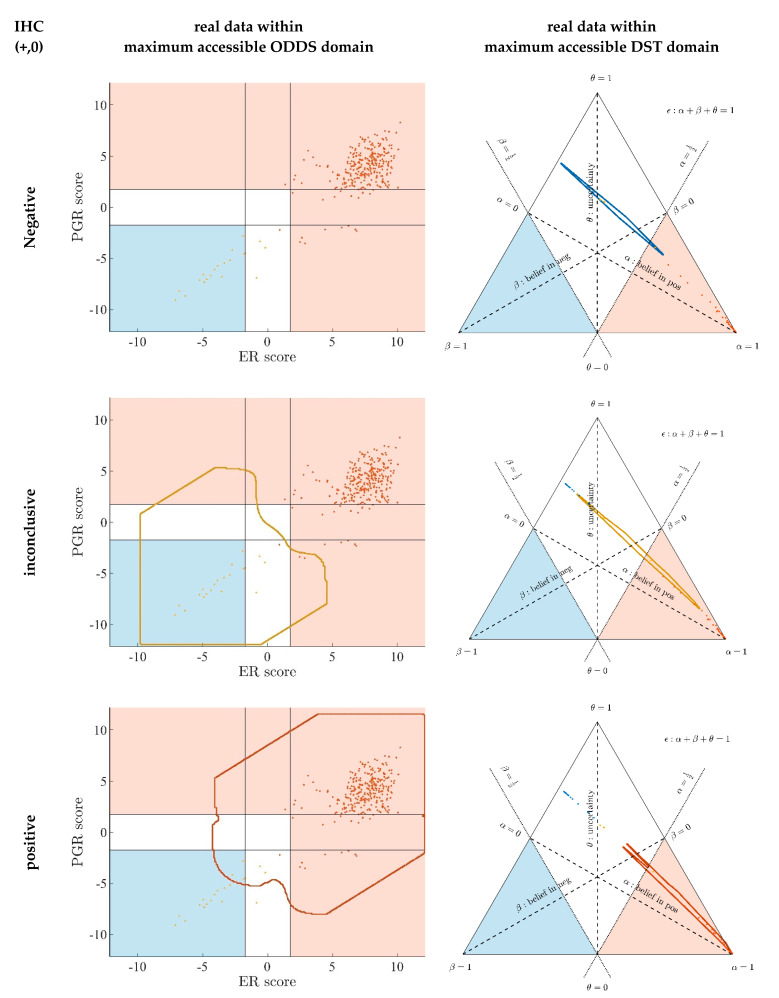
Real sample data within maximum accessible domains for the IHC case = (+,0). For general features of display see caption to Figure A3.

## Data Availability

All data were downloaded from Gene Expression Omnibus.

## Appendix A

### Appendix A.1. Download and Cleansing of Data

The Gene Expression Omnibus (GEO) [88,89] was screened for breast cancer studies using the Affymetrix chip U133A+2.0 [90] and found 38 studies. CEL files and clinical data (characteristics), such as estrogen and progesterone receptor statuses (ER, PGR) and HER2, all measured by immunohistochemistry (IHC) were downloaded and curated to arrive at a clean database [91,92], already described in our previous work [37]. Data cleansing meant, in particular:

- Only tumor samples were considered, controls excluded;
- Only tissue samples were considered, cell lines excluded;
- Replicates were removed;
- All samples were pairwise checked for being duplicates. CEL files with equal medical data (expression, clinical) may differ, just in format or container packing. Hence, actual expression values needed to be compared to safely locate duplicates;
- If duplicates in expression data were found to differ in metadata, these were curated manually;
- Some GSE studies have been ‘enriched’ with samples from other (previous) GSE studies. Such samples become duplicates if both of these studies were evaluated in combination. We always left such samples with their original study and removed its duplicate from the later GSE study;
- We detected damaged samples by RMAexpress [93] and removed them;

After cleansing, 3753 samples remained to be used for joint evaluation [94].

A plethora of normalization methods for microarrays has been proposed [51,95,96,97,98], as evaluated by Bolstad [57]. Based on the results of our previous work [99], we performed RMA, using the implementation MATLAB affyrma. We had also evaluated several types of batch corrections [100,101,102,103] (Luo et al., 2010, Leek et al., 2012, Müller et al., 2016, Johnson et al., 2007) and surrogate variable analysis (SVA) [104,105,106] (Leek and Storey, 2007, Leek et al., 2017, Parker et al., 2014) for combined microarray studies. However, no clear benefit of explicit batch corrections could be clearly demonstrated [99]. Therefore, we preferred to perform ‘global RMA normalization‘, over all studies being combined.

### Appendix A.2. Selecting HER2 Negative Patients

Next, HER2-negative patients were selected as follows. Patients with positive IHC estimates for HER2 (${\mathrm{HER}2}_{\mathrm{IHC}}^{+}$) were excluded right away and samples with negative HER2 (${\mathrm{HER}2}_{\mathrm{IHC}}^{-}$) retained. For patients with missing IHC estimates for HER2, we attempted imputation via gene expression, using the ODDS method [37] (see also Table A3). Only ‘safe’ imputations, yielding ${\mathrm{HER}2}_{\mathrm{IHC}}^{-}$, were retained, in accordance with our previous work [37], yielding 2519 patients in all. The final set of studies considered is listed in Table A1 of Appendix A.1.

**Table A1:** Microarray studies used. *N*: number of samples. All: total number of samples in study. ER_IHC_: number of samples with IHC measurement of estrogen receptor status. PGR_IHC_: likewise for progesterone. For reference see also our previous work [37,59,99].

		*N*		
Study	City	All	ER_IHC_	PGR_IHC_
GSE5460	Boston	17	17	0
GSE6532	Toronto	78	78	77
GSE12276	Rotterdam	118	0	0
GSE16391	Toronto	50	50	50
GSE16446	Toronto	84	84	0
GSE18728	Seattle	15	15	14
GSE18864	Lyngby	60	60	60
GSE19615	Manhattan	79	79	79
GSE20685	Taipei	163	0	0
GSE20711	Toronto	52	52	0
GSE22035	SAINT-CLOUD	27	27	0
GSE23177	Leuven	80	80	0
GSE26639	Paris Cedex 05	144	144	142
GSE27120	Brussels	26	26	26
GSE29431	Barcelona	23	23	23
GSE31448	Marseille	286	286	271
GSE36771	Auckland	86	86	85
GSE42568	Dublin	65	63	0
GSE43358	Brussels	43	43	43
GSE43365	Boston	98	98	98
GSE46222	Washington	26	26	0
GSE47389	Rotterdam	47	47	47
GSE48390	New Taipei City	34	34	0
GSE48905	Hørsholm	20	20	0
GSE50567	Gliwice	25	25	0
GSE58792	New York	33	33	0
GSE58812	Saint Herblain	107	107	107
GSE61304	Singapore	41	38	33
GSE65194	PARIS	70	67	41
GSE71258	Missouri	94	77	77
GSE76124	Houston	198	198	198
GSE76274	Houston	44	44	44
GSE87007	Brussels	24	24	24
GSE88770	Brussels	108	108	107
GSE95700	Taipei City	54	54	54
∑		**2519**	**2213**	**1700**

### Appendix A.3. Selecting Genes and Probe Sets for Estrogen and Progesterone Receptors

Several genes are mentioned in the literature [22,107] to be relevant for estrogen and progesterone. In addition, we used the limma-package [55] to screen CEL files (of those 2519 HER2-negative patients) for probe sets, discriminating between positive and negative IHC statuses, separately for ER and PGR, and sorted results by ascending *p*-values. After mapping back from probe sets to genes, we finally adopted the very receptor gene and one co-gene, in addition, for ER and PGR, respectively. For details, please refer to our previous work [58], and Table A2.

**Table A2:** Probe sets and logistic regression for receptor genes and co-genes. $c_{0}$ and $c_{1}$ are coefficients from logistic regression for IHC values (dependent variable) versus gene expression (independent variable) [37,58]. Probe sets refer to the Affymetrix chip U133A + 2.0. For ‘deviance of fit’, see p. 118 in McCullagh [108]. Upper limits for beliefs ($\hat{\alpha }$, $\hat{\beta }$) are explained in Appendix A.4 and Appendix A.5. See Equations (A1) and (A2) for computing the limits, and Equation (4) for their application.

				Logistic Regression Parameters		Logistic Regression Quality		Upper Limits for Beliefs	
			Probe Set	$c_{0}^{}$	$c_{1}^{}$	Deviance of Fit	Number of Samples	$\hat{\alpha }$	$\hat{\beta }$
estrogen	gene	ESR1	205225_at	9.905	−1.061	1086.6	2213	0.814	0.887
	co- gene	AGR3	228241_at	5.582	−0.710	1253.1		0.794	0.840
progesterone	gene	PGR	208305_at	7.449	−0.983	1107.4	1700	0.753	0.702
	co- gene	ESR1	205225_at	8.617	−0.834	1249.8		0.618	0.817

**Table A3:** Probe sets and logistic regression for gene and co-gene of HER2. $c_{0}$ and $c_{1}$ are coefficients from logistic regression for IHC (dependent variable) versus gene expression (independent variable) [37,58]. Probe sets refer to the Affymetrix chip U133A + 2.0. For ‘deviance of fit’, see p. 118 in McCullagh [108].

				Logistic Regression Parameters		Logistic Regression Quality	
			Probe Set	$\beta_{0}^{GE}$	$\beta_{1}^{GE}$	Deviance of Fit	Number of Samples
HER2	gene	*ERBB2*	216836_s_at	15.963	−1.408	1421.2	2430
	co-gene	*PGAP3*	221811_at	17.756	−2.168	1330.6	

### Appendix A.4. Tailoring Beliefs in Receptor Gene Expression to a Given Accuracy of IHC

It is intuitively understandable that such an upper limit for the belief in ‘positive’ must relate to true and false positive rate (*TP*, *FP*), as well as true and false negative rate (*TN*, *FN*) of the measuring process in question. It was one of the main achievements in our previous work [37], to coin this qualitative argument into the following Equation:

(A1) $${\hat{\alpha }}_{\mathrm{Expr}}=\frac{TP\cdot TN-FP\cdot FN}{\left(TP+FP\right)\cdot \left(TN+FP\right)}$$

(A2) $${\hat{\beta }}_{\mathrm{Expr}}=\frac{TP\cdot TN-FP\cdot FN}{\left(TN+FN\right)\cdot \left(TP+FN\right)}$$

${\hat{\alpha }}_{\mathrm{Expr}}$ and ${\hat{\beta }}_{\mathrm{Expr}}$ quantify the remaining doubt, even if measurements seem perfectly clear (maximum gene expression). *TP*, *FP*, *TN* and *FN* can be obtained from the discrepancies between IHC and the prognosis obtained from gene expression, according to conventional statistics, using a cut-point of 0.5 in the logistic regression.

### Appendix A.5. Formulating IHC Data in Terms of Evidence

Gene expression, $x_{\mathrm{Expr}}$, is a continuous variable, and so is evidence derived thereof: $\alpha_{\mathrm{Expr}}\left(x_{\mathrm{Expr}}\right),\beta_{\mathrm{Expr}}\left(x_{\mathrm{Expr}}\right),\theta_{\mathrm{Expr}}\left(x_{\mathrm{Expr}}\right)$, as shown in Figure 2. Opposed to that, IHC yields binary results (+/−) and, hence, evidence thereof are constants, one set for a positive IHC result ($\alpha_{{\mathrm{IHC}}^{+}}$ $\beta_{{\mathrm{IHC}}^{+}}=0$ $\theta_{{\mathrm{IHC}}^{+}}$) and a second set for a negative IHC result ($\alpha_{{\mathrm{IHC}}^{-}}$, $\beta_{{\mathrm{IHC}}^{-}}$, $\theta_{{\mathrm{IHC}}^{-}}$). How shall these values be chosen?

For a start, we draw on the following findings: Quality assessments of IHC [38,39] revealed that approximately 85% of IHC estimates can be assumed to be correct and, consequently, 15% to be false [40,41,42].

To implement these findings in terms of DST, we first consider all *IHC measurements with positive outcome*, as illustrated in Figure A2, upper panel. Among these, some have resulted true positive, by quality of the measuring method, others resulted true positive by chance. Both taken together make up the (total) number of true positives (*TP*), i.e., 85% of all positive outcomes, according to the above data from the literature. The remaining 15% of positive IHC outcomes represent wrong results, namely false positives (*FP*), i.e., samples negative in reality. We may now assume (on good grounds) that 15% is also a reasonable estimate for the fraction of samples being true positive by chance, not by quality of the method, see Figure A2, upper panel.

Accordingly, given a positive IHC measurement (IHC^+^), the total evidence comes about as follows:

- Due to the positive IHC measurement, there is no evidence at all for the status being (truly) negative due to quality of the method, hence $\beta_{I{\mathrm{HC}}^{+}}=0$.
- Being measured as true positive by chance or as false positive by error represents all measurements not being true by quality of the method. Together they make up 30%, represented by $\theta_{I{\mathrm{HC}}^{+}}=0.3$. We assume that these split in equal parts into 15% true positive by chance and 15% false positive by error.
- Hence, cases being true by quality make up the remaining 70%, represented by $\alpha_{I{\mathrm{HC}}^{+}}=0.7$.
- Since all items add up to 1 (Equation (2)), we obtain $\beta_{{\mathrm{IHC}}^{+}}=0$, and the whole evidence after a positive IHC result is ($\alpha_{{\mathrm{IHC}}^{+}}=0.7$, $\beta_{{\mathrm{IHC}}^{+}}=0$, $\theta_{{\mathrm{IHC}}^{+}}=0.3$).

On the contrary, after a *negative* IHC measurement, ${\mathrm{IHC}}^{-}$, we obtain the evidence: $\alpha_{{\mathrm{IHC}}^{-}}=0.0$, $\beta_{{\mathrm{IHC}}^{-}}=0.7$ and $\theta_{{\mathrm{IHC}}^{-}}=0.3$, see panel (b) of Figure A2.

### Appendix A.6. Ternary Plots Reflect Subgroups within Patient Cohort

After introducing the more general features of ternary plots in Section 2.2.4, we now describe specific features of actual patient data of this study within this framework, see also Figure 4. Considering ***just one gene plus IHC*** as evidence, it is easy to make subgroups of patients transparent, a possibly valuable feature for personalized medicine, illustrated by the following features:

- Evidence for patients is not distributed evenly all over the ‘triangle plain of evidence’, but samples are grouped in ‘traces’, which deserves explanation: first, we note that exactly *three* lines appear and each sample belongs to one of these lines; no sample is found apart. The fact that we deal with three possible states of IHC values (+, −, inc) already points towards a possible reason, and this is in fact true: it is varying IHC statuses, which give rise to these lines. Suppose that, for a given IHC status, e.g., positive, we consider different values of gene expression, *x*_Expr_. When computing corresponding evidence, $\alpha \left(x_{\mathrm{Expr}}\right),\beta \left(x_{\mathrm{Expr}}\right),\theta \left(x_{\mathrm{Expr}}\right)$, these will appear along a straight line. This is visually obvious but can, in fact, be formally proven mathematically, resorting to Equations (1), (4) and (6). Hence, each of the specific lines may be labeled, accordingly (${\mathrm{ER}}_{\mathrm{IHC}}^{+}$, ${\mathrm{ER}}_{\mathrm{IHC}}^{-}$ and ${\mathrm{ER}}_{\mathrm{IHC}}^{\mathrm{inc}}$), see Figure 4a.
- Note also that the red line of ${\mathrm{ER}}_{\mathrm{IHC}}^{+}$ samples starts near the corner *α* = 1, but *not exactly at* the corner: even a positive IHC and large gene expression cannot guarantee a positive prediction—some small uncertainty (*θ*) remains. At the same time, for such a sample, there is no evidence whatsoever for a negative status. Hence *β* = 0, and the line starts at the ternary plot’s side representing *β* = 0. Such a sample represents the total opposite to the lower left corner—where *β* = 1 (surely negative).
- After originating close to the lower right corner of (marked with *α* = 1) the line for ${\mathrm{ER}}_{\mathrm{IHC}}^{+}$ (red), proceeds across the sub-area indicating receptor positive (shaded red). These samples have ${\mathrm{ER}}_{\mathrm{IHC}}^{+}$ status (all dots, no circles), being confirmed by gene expression, ending up as positive predictions. After crossing the decision border at *α* = 0.5, this line still represents samples with ${\mathrm{ER}}_{\mathrm{IHC}}^{+}$, which has obviously been questioned by gene expression; hence, prediction was rendered ‘inconclusive’ according to DST (samples lie within the kite-shaped area). Coloring these samples, according to ODDS, most vividly reveals differences in prediction: although located within the DST-inconclusive region, ODDS predicts some of these samples as positive, the majority as inconclusive (i.e., agrees with DST), but a few as negative (see the blue dots towards the end of the line in the upper left).
- Note that lines for ${\mathrm{ER}}_{\mathrm{IHC}}^{+}$ and ${\mathrm{ER}}_{\mathrm{IHC}}^{-}$ never protrude into the opposing definite areas, for the following reason: given ${\mathrm{ER}}_{\mathrm{IHC}}^{+}$, gene expression can by no means reverse the prediction to surely negative. At the most, it may downgrade it to inconclusive. The same is true for ${\mathrm{ER}}_{\mathrm{IHC}}^{-}$. The white, kite-shaped area segregates the areas of positive and negative predictions, which is reasonable.
- Only at one single point, two strongly opposing items of evidence might, in principle, become close to one another (at the point *α* = *β* = 0.5, along the baseline of the ternary plot, see the tutorial Section 2.2.4 for further discussion). As a matter of fact, such samples do not occur in reality (in our cohort), and both lines meet farther outside, within the inconclusive region. In other words, if evidence incorporates contradiction, DST renders them inconclusive—as a precaution.
- Finally, the line for ${\mathrm{ER}}_{\mathrm{IHC}}^{\mathrm{inc}}$ crosses the whole decision triangle, from surely positive (right side) through the inconclusive region (mid), towards surely negative (left side). Since no IHC status is available for these samples (shown as circles), gene expression is free to render this ample range of predictions.

The characteristics of ternary plots, enhanced data interpretation and its relevance for personalized medicine, have been introduced along a simple example—featuring only IHC status and the expression of one single gene—in order to be intuitively clear. In the following, the ‘full’ model (including co-genes), will be evaluated along the very same conceptual lines.

### Appendix A.7. Evidence Patterns for Subsets of Patients

We have already demonstrated (Section 2.2.4 and Figure 4) for a single gene and IHC (as the only sources of evidence) that conspicuous arrangements of data points are rooted in the IHC status: for all patients with a given IHC status, evidence was seen to lie on straight lines, see Figure 4. Now, considering four genes (two receptor genes, two co-genes), the situation becomes more complex. More degrees of freedom in the input variables penetrate into the final prediction, and the lines (as seen for single genes) expand to lengthy polygons.

To scrutinize the underlying mechanism, we first display data separately for distinctive IHC statuses, e.g., for IHC = (−, −) see Figure A3. Again, we display samples in ODDS coordinates (left column) side by side with DST ternary coordinates (right column). In the left panels, the locations of samples indicate their prediction according to ODDS, while their color indicates their prediction according to DST, and vice versa. Again, differences in prediction are read off easily. Note that the very same, specific subset of samples (IHC = (−, −)) is shown in all panels of Figure A3.

The following questions then arise: Do absolute, distinct boundaries exist for the evidence of samples with given IHC statuses? If yes, where are they located? To find out, we computed so-called ‘maximum accessible prediction domains’ (MPDs) as follows: artificial (simulated) samples were generated by scanning each gene and co-gene over the entire domain of measured expression values (in our data, 2.3 to 15.2) in 100 equidistant points, yielding 100^3^ = 10^6^ generated samples. For each generated sample, we computed predictions by both, ODDS and DST, and plotted them into the ODDS plane and the ternary triangle, respectively. These predictions spread out over much larger areas than the samples of actual patients did, and we, hence, termed these areas ‘maximum accessible prediction domains’ (MPD). Rather than showing all samples together, we displayed them separately for each prediction (negative, inconclusive or positive); see the rows ‘negative’, ‘inconclusive’ and ‘positive’ in Figure A3. Using 10,000 simulated samples, MPDs would be scrammed with points when being plotted. We do not display all of them (would look like filled areas) but only show the outline of these areas. In each row of Figure A3, the left panel shows the MPD of DST, arranged in ODDS coordinates. Vice versa, the right panel shows the MPD of ODDS in DST ternary coordinates. Note the following:

- Since an MPD represents a maximum area, no sample of the same color appears outside, e.g., no blue sample (predicted negative by DST) may lie outside the blue MPD in the left panel of Figure A3.
- No blue sample (predicted negative by ODDS) may lie outside the blue MPD in the right panel of Figure A3.
- While predictions coded in color transgress decision borders according to location, they never leave the maximum accessible prediction domain of their own prediction method.
- Samples of real patients were never seen to yield contradicting predictions (e.g., negative by DSST and positive by ODDS), but MPDs well intrude into contradicting domains. For example, the negative MPD of DST (outlined blue) not only reaches into the inconclusive region, but well overlaps, even with the positive area of ODDS (Figure A3, left column, row 1). A second example is the positive MPD of ODDS (outlined red), penetrating into the decisively negative domain of DST (Figure A3, right column, row 3).
- Note that these ‘contradicting’ overlaps are rooted in extreme expression values, occurring in generated samples only, but have never been seen in our real data. Thus, these potential areas of contradiction between ODDS and DST remain a theoretical possibility to be considered, which does not infringe, however, application of these methods to data of real studies.
- Note that the dots (evidence) of these 10,000 simulated samples are not evenly distributed over the MPD. This is similar to the evidence of real samples; these also appear in fairly restricted zones, well within the respective MPD. One could generate 2-dimensional histograms, showing the density of these simulated samples.

Other IHC statuses are covered in Figure A4 (+, +), Figure A5 (+, −) and Figure A6 (0, 0). More cases are shown in the Appendix, see Figure A7 (−, 0) and Figure A8 (+, 0).
